# Spatial metabolic and phenotypic characterization of the germ‐free mouse model

**DOI:** 10.1111/nyas.70002

**Published:** 2025-08-04

**Authors:** Lauren Adams, Heather Hulme, Clio Dritsa, Connor Lynch, Vicky Taylor, Orhan Rasid, Richard Burchmore, Richard J. A. Goodwin, Daniel M. Wall

**Affiliations:** ^1^ School of Infection and Immunology, College of Medical, Veterinary and Life Sciences University of Glasgow Glasgow UK; ^2^ Integrated Bioanalysis, Clinical Pharmacology and Safety Sciences BioPharmaceuticals R&D, AstraZeneca Cambridge UK; ^3^ Biological Services Facility The University of Manchester Manchester UK

**Keywords:** germ‐free, imaging, microbiome, molecules, spatial biology

## Abstract

The gut microbiome has been strongly linked to health and disease, exerting its systemic effects through host and bacterial molecules that disseminate from the intestine. Understanding how these molecules may contribute to, exacerbate, or even improve specific health conditions is a key mechanistic challenge in microbiome research. Using the germ‐free mouse model, we used a spatial biology approach to map the location of small molecules in intestinal and systemic tissues in addition to phenotyping cells in their vicinity. Significant differences were noted in molecular species across all tissues tested, including the ileum, colon, spleen, lung, liver, and kidney, with the greatest number of changes in the liver. Molecules putatively identified as phenol sulfate and 5‐amino valeric acid betaine were noted to have significantly changed in abundance in the germ‐free mouse intestine as well as systemically. Phenotypic characterization of germ‐free mouse organs identified significant alterations in immune cell numbers indicative of an aberrant immune response, underlining the critical role of the microbiome in immune stimulation and priming, even at sites distal from the intestine. Our findings highlight the significant molecular and cellular changes that occur in the absence of a gut microbiota, identifying key microbiome‐derived metabolites and host phenotypic signatures.

## Graphical abstract

The germ‐free mouse model is a useful tool to understand the influence of the gut microbiota on host physiology. Using spatial metabolomics approaches we mapped the systemic location of small molecules in germ free and colonized mice, focusing on the intestine, spleen, lung, liver, and kidney. By combining this metabolite tracking with systemic phenotypic characterization of cells in their vicinity through imaging mass cytometry, we could identify significant alterations in immune cell numbers indicative of an aberrant immune response. This underscored the critical role of the microbiome in immune stimulation and priming, even at sites distal from the intestine.

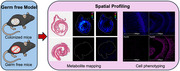

## INTRODUCTION

Microbial colonization of the intestinal tract is essential for the maturation of the intestine and the immune system and for physical and metabolic protection from pathogens.^1^, ^2^ The gut microbiota undergoes continuous compositional changes until children reach between 2 and 5 years of age when it reaches a sufficient level of diversity which is maintained into adulthood and is represented broadly by bacteria of the genera *Bacteroides*, *Prevotella*, *Ruminococcus*, and *Clostridium*.^3^, ^4^ Alterations in the intestinal microbiota in early infancy increase the risk of childhood obesity, type 1 diabetes, nonalcoholic fatty liver disease (NAFLD), and autoimmune conditions.^5^, ^6^

This link between dysfunctional microbiota and the development of disease involves significant crosstalk with the immune system.^7^ Commensals can suppress acute inflammation in murine models with the microbially derived molecules butyrate and trimethylamine oxide driving monocyte‐to‐macrophage differentiation or macrophage polarization.^8^, ^9^ When innate immune cells encounter microbially derived ligands or specific bacteria, cytokines are secreted to signal the differentiation of naive T cells of the adaptive immune response into Tregs, Th1, Th2, or Th17 cells.^10^, ^11^ Treg cells can be anti‐inflammatory as they suppress the activation of mast cells, basophils, and eosinophils, whereas Th cells can direct and amplify inflammatory responses.^12^, ^13^ Therefore, this balance of T cells, influenced by the gut microbiota, is a critical factor for immune homeostasis and can prevent the development of inflammatory, autoimmune, and allergic diseases. Additionally, dendritic cells (DCs) exposed to bacterial antigens can prime and activate T cells which when activated can mediate their diverse array of effects.^14^

The gut microbiome also influences the immune status and function of distal organs.^15^ It is difficult for bacteria to disseminate systemically due to the existence of the gut–vascular barrier which prevents bacteria from entering the portal circulation.^16^ Therefore, the majority of microbes interact with the host in an indirect manner mediated by their metabolic products that can cross barriers.^15^, ^17^ The complex community of microbes that colonize the human intestine have a huge capacity for metabolite production, which plays a fundamental role in host physiology.^18^ In particular, gut‐derived metabolites such as short‐chain fatty acids, amino acids, and bile acids can directly affect hepatic glucose and lipid metabolism, and depletion of the gut microbiota results in dysfunctional hepatic lipid accumulation, which in turn can impair organ function.^18^, ^19^ Gut–liver communication mediated by microbial metabolites has been implicated in various liver diseases such as gallstones and NAFLD.^20^, ^21^ The dissemination of microbial metabolites is likely to also affect the function and immune profile in other organs such as the spleen, kidneys, and lungs, but this has yet to be examined in detail.

This study aimed to identify microbial molecules and host molecules under microbial influence that are systemically disseminated and evaluate their role in immune cell function at those sites. Using a germ‐free (GF) mouse model, these metabolites were spatially localized and their relative abundance was quantified using mass spectrometry imaging (MSI), and their potential role in altering the phenotype of neighboring cells was determined using imaging mass cytometry (IMC). Small molecules or metabolites play an important role in immune cell function, inflammation, and cell signaling, thus identifying metabolites that are altered due to microbial colonization and evaluating their role in cell functioning might elucidate mechanisms involved in microbe–host interplay that impact mammalian physiology.

## MATERIALS AND METHODS

### Animals

Six‐ to eight‐week‐old C57BL/6J GF and specific pathogen‐free (SPF) mice were sourced from the University of Manchester, Gnotobiotic Facility. Both GF and SPF mice were fed the same pelleted diet, which was sterilized by irradiation with 50 kGy. Animals were kept under a 12‐h light/12‐h dark cycle. The Manchester Gnotobiotic Facility was established with the support of the Wellcome Trust (097820/Z/11/B) using founder mice obtained from the Clean Mouse Facility, University of Bern. GF mice were of mixed population (*n* = 4 female, *n* = 2 male) and SPF were all female (*n* = 6). Animals were sacrificed via CO_2_ asphyxiation followed by the cutting of the femoral artery. After sacrificing mice, the entire intestine was taken, colon and ileum contents were gently removed, and it was cut longitudinally along the mesenteric line and swissed rolled before embedding it into hydropropyl‐methylcellulose/polyvinylpyrrolidone hydrogel. Tissue was then snap‐frozen in a slurry of dry ice and isopropanol before rinsing in a slurry of dry ice and isopentane for 30 s. Frozen blocks were then left on dry ice to allow alcohol to evaporate before storing at −80°C. Other tissue types including liver, kidney, spleen, and lung were removed, frozen, and stored as described.

Ten micrometers thick sections were cut from frozen tissue blocks using a cryostat microtome (Thermo Scientific). Consecutive cutting of sections was repeated until enough sections were obtained, and these were thaw‐mounted onto Superfrost nonconductive microscope slides for desorption electrospray ionization (DESI)‐orbitrap‐MSI. All slides were dried, vacuum packed, and stored at −80°C following sectioning. During tissue preparation, one SPF liver and one SPF lung sample were damaged, so for these organs, the SPF group has five samples instead of six.

### Desorption electrospray ionization mass spectrometry imaging

Vacuum‐sealed slides were brought to room temperature (RT) before opening, and all DESI‐MSI experiments were set up as follows. DESI‐MSI was performed on a ThermoFisher Scientific Q‐Exactive mass spectrometer equipped with an automated Prosolia 2D DESI source. A home‐built DESI sprayer assembly was used with the spray tip positioned at 1.5 mm above the sample surface and at an angle of 75°. The distance between the sprayer to mass spectrometer inlet was 7 mm with a collection angle of 10°. The spray solvent was methanol/water (95:5 v/v), delivered at 1.5 µL/min using a Dionex Ultimate 3000 pump (ThermoFisher Scientific) at a spray voltage of 4.5 kV. Nitrogen was used as the nebulization gas at a pressure of 7 bars. The Q‐Exactive mass spectrometer was operated in positive ion and negative ion mode for all analyses using an S‐Lens setting of 75 V. To acquire full mass spectra in the mass range of *m/z* 100–1000, a mass resolution of 70,000, a target of 5,000,000, and an injection time of 150 ms were used. The .raw files were converted into mzML files using ProteoWizard *msConvert* and compiled into .imzML format using *imzML converter* version 1. Data sets were then converted into .slx files and analyzed using *SCiLS Lab MVS* (version 2020b Premium 3D). All DESI data analyses were performed on root mean square normalized data. Relative operating characteristic (ROC) curve analysis was performed to visualize the discrimination capability of an *m/z* value between two conditions. Area under the curve (AUC) values were generated by ROC analysis and cut‐off ranges were set at AUC >0.75 and AUC <0.25 to produce a list of *m/z* that might discriminate between conditions. Spatial resolution for DESI analysis was optimized for different tissue types based on the resolution required for finer tissue structures while limiting analysis time to prevent metabolite changes during running. Gut tissue was captured at 60 µm resolution and all other tissues at 100 µm.

### Multivariate and univariate analysis

Following ROC analysis, unsupervised and supervised multiclass classification and correlation were performed to visualize data and highlight molecules of interest. This multivariate analysis (MVA) was performed using the SIMCA 17 software package (Sartorius Stedim Biotech). Unsupervised principal component analysis (PCA) was applied to obtain an overview of data and detect any potential outliers. Subsequently, supervised partial least squares‐discriminate analysis (PLS‐DA) was used to identify specific changes among the groups. In PLS‐DA, the Y variable was assigned to a defined class and corresponded to the X variable. The model was generated after the data was autofitted according to *R* ^2^, *Q* ^2^, and classification performance. The quality of the model was validated using two parameters: R^2^Y(cum) (goodness of fit) and Q^2^(cum) (goodness of prediction). A threshold of >0.5 is widely accepted as a good model classification to assure reliable predictive capabilities. Group separation is presented as score plots. Variable importance in projections (VIP) was used as a readout from PLS‐DA that reflects the metabolites contribution to the model. VIP >1 indicates a higher percent variation in the model when a metabolite is included, whereas VIP <0.5 indicates that a metabolite plays a less important role. Following MVA, univariate analysis was performed in GraphPad prism (8.4.3). The measurement data were expressed as mean relative abundance ± SD. A nonparametric *t*‐test with a 5% false discovery rate correction was used for comparing groups. Statistical significance was defined as a *p*‐value less than 0.05. Molecules with a VIP >1 and/or a *p*‐value <0.05 were selected for further analysis.

### Histological staining

After DESI‐MSI, tissue sections were fixed by submerging them in 4% paraformaldehyde for 10 min. Sections were then stained with Mayer's hematoxylin for 1 min, before rinsing with tap water and submerging in acid alcohol. The tissue was then stained with eosin for 20 s, followed by rinsing with tap water and washed three times with absolute ethanol. Tissue sections were then submerged in xylene for 1 min and coverslips were applied using dibutylphthalate polystyrene xylene mountant. Hematoxylin and eosin (H&E) stained tissue was imaged with an Aperio CS2 digital pathology scanner (Aperio Tech) at 40× magnification and observed in ImageScope software (Aperio Tech).

### Identification of metabolites

Molecules that were significantly altered were putatively identified using the Human Metabolome Database (HMDB) based on the mass accuracy provided by DESI‐MSI analysis. Searches were focused on mass (M) ± most common adducts—negative mode included M^−^ hydrogen (H) and M^+^ chlorine (Cl), positive mode included M^+^H, M^+^ potassium (K), and M^+^ sodium (Na). To further identify molecules, DESI‐Tandem MS (MSMS) was performed on tissue sections using a ThermoFisher Scientific Q‐Exactive Orbitrap mass spectrometer. The data were acquired in either positive or negative mode with a spray voltage of 4.5 kV, 70,000 resolution, maximum injection time of 1000 ms, and S‐lens settings of 75 V. Precursor ion mass was set with a mass tolerance of 0.5 *m/z* and the mass range acquired was optimized for each analyte. Higher energy collision dissociation with normalized collision energy (NCE) was used for the fragmentation of ions during MSMS measurements ranging from 10 to 50 NCE. The identities of the ions were established based on the product ion spectra after comparing them to spectra from previously published data using online platforms, HMDB and mzCloud (https://www.mzcloud.org/).

### Metabolite enrichment analysis

Significantly changed molecules are shown as clustered heatmaps generated by the online MetaboAnalyst 5.0 platform (https://www.metaboanalyst.ca/). All available metabolite identities were used in MetaboAnalyst metabolite set enrichment analysis. This tool detects major pathways that are associated with the metabolites present in the study. The applied library was the Kyoto encyclopedia of genes and genomes (KEGG) human metabolic pathways, comprising 84 metabolite sets. The enrichment ratio was defined as the ratio of observed hits (detected metabolite) per pathway to the count expected by chance. One‐tailed *p*‐values were provided after adjusting for multiple testing, enrichment was considered significant when *p*<0.05.

### Imaging mass cytometry

Tissue sections were fixed using 4% paraformaldehyde for 10 min at RT. This was followed by tissue permeabilization with 1× casein solution containing 0.1% Triton X‐100 for 5 min at RT. Tissue sections were then incubated with blocking buffer (1× casein solution) for 30 min at RT inside a humidified chamber. An antibody cocktail was prepared with the appropriate dilution of antibodies (Table S1). Tissues were then fully covered with antibody solution and incubated overnight in the humidifier chamber at 4°C. DNA Ir‐Intercalator (Fluidigm) was diluted 1:400 using phosphate‐buffered saline (PBS) and pipetted onto tissue, followed by a 30‐min incubation at RT. The tissue was washed in PBS for 5 min and this was repeated three times after each step of the protocol. After the final PBS wash, the tissue was rinsed for 30 s in deionized water and left to dry and stored at RT before imaging.

Tissue was imaged using the Hyperion Imaging System (Fluidigm), rasterizing at 200 Hz with an ablation energy of 5 dB. The laser was frequently tuned between imaging runs to ensure tissue was fully ablated without leaving scratches on the glass. Once the acquisition was complete, images were exported from MCDviewer as a 32‐bit TIFF and imported into the HALO image analysis platform (Indica Laboratories) for analysis. Using a random forest machine learning tissue classifier module, the colon and ileum were segmented into muscularis and mucosa, while the liver and lung were analyzed as whole tissue and vessels based on morphology and blood vessel cell marker, CD31. The Hiplex module was first used to segment the DNA intercalator from each cell with a proxy for the cytoplasm being 1 µM radius from the nucleus. Thresholds were then set to define positive cell staining for each marker. Phenotypes for cells of interest were defined using markers cited in the current literature (Table S2). The percent of positive cells and percent of positive phenotype cells were defined within tissue regions. Statistical analysis was performed in GraphPad prism, and comparisons were made between the GF and SPF mice using two‐tailed *t*‐tests. Differences between disease and control samples were considered significant when *p*<0.05.

### Statistical analysis

**FIGURE 1:**
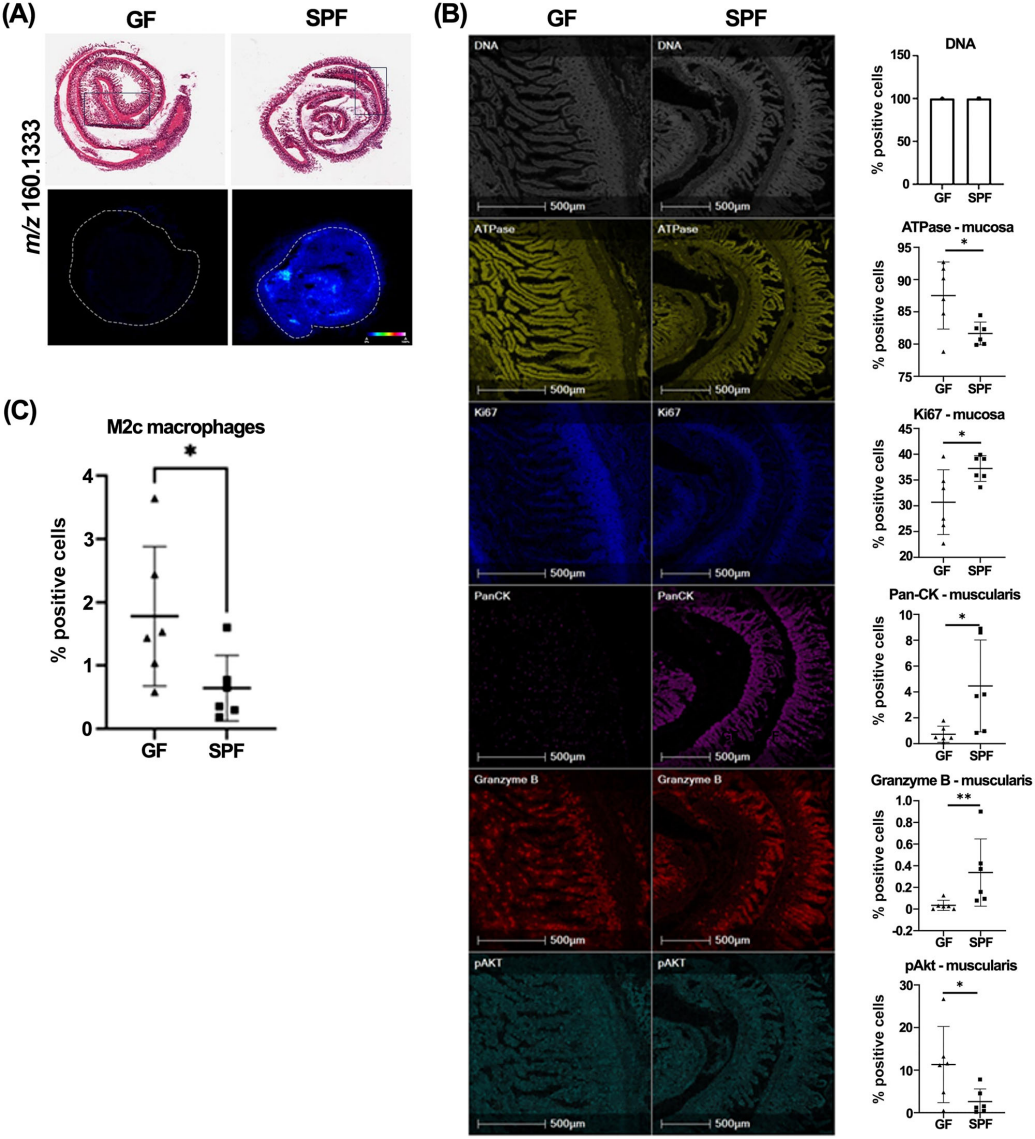
MSI of *m/z* 160.1333 abundance in the ileum and IMC of biological markers of cell function in the neighboring ilea mucosa and muscularis. (A) H&E stained sections with rectangular boxes indicating the region selected for IMC and MSI heatmap of *m/z* 160.1333, with color bar showing 0−100% relative abundance. (B) Representative IMC images from the region indicated in (A) for GF and SPF groups and percent of cells positive for that marker. From top to bottom, DNA intercalator identified single cells within the tissue section, ATPase, Ki67, pan‐CK, granzyme B, and pAKT. (C) Graph showing the percentage of cells with a phenotype indicative of M2c macrophages. Graphs show mean ± standard error of the mean (SEM) and show four or five biological replicates. For normally distributed data, a two‐tailed *t*‐test was used to determine statistical significance. For granzyme B data, which was not normally distributed, a Mann−Whitney U test was applied. *p<0.05 and **p<0.01 were defined as statistically significant.

For assessing statistical significance between GF and SPF groups, all data were first assessed for normality using the Shapiro−Wilk test. Where data were normally distributed, a two‐tailed *t*‐test was used to determine statistical significance. For data not normally distributed, a Mann−Whitney U test was applied; *p*‐values <0.05 were defined as significant. Full details of statistical analysis relating to Figures 1, 3, and 6 are included in Table S3.

## RESULTS

### Metabolic and cellular alterations in the ileum of GF compared to SPF mice

MSI was used to investigate the molecular changes in the ileum of SPF and GF mice. ROC analysis found one peak of *m/z* 160.1333, which had increased 14.73‐fold (*p*<0.0001) in the SPF ileum, and could discriminate between the two groups (Figure 1A). The molecule was putatively identified as 5‐AVAB using the HMDB, but MSMS was not able to accurately confirm this identity, and our own and others’ work has highlighted the existence of multiple microbial metabolites at this *m/z*.^22^, ^23^ As only a single molecule of interest was identified through ROC analysis, MVA could not be performed.

IMC was performed at 1 µM spatial resolution on adjacent tissue regions (highlighted by blue rectangular boxes in Figure 1A) revealing changes in structural and immune protein markers (Figure 1B). In the mucosa when comparing SPF to GF mice, the percentage of ATPase^+^ cells decreased (1.07‐fold; *p* = 0.0256), whereas the percentage of Ki67^+^ expressing cells increased (1.21‐fold; *p* = 0.0394). M2c macrophages, a subgroup of M2 macrophages that are F4/80^+^ and CD163^+^, decreased 2.67‐fold (*p* = 0.0453) in the mucosa of SPF compared to GF mice. In the muscularis, pan‐CK^+^ and granzyme B^+^ expressing cells increased 6.09‐fold (*p* = 0.0297) and 9.78‐fold (*p* = 0.0400), respectively, in SPF mice, while pAKT^+^ expressing cells decreased (4.31‐fold; *p* = 0.0477). However, IMC did not identify significant changes in specific cell phenotypes in the muscularis between the two groups. However, the presence of gut microbiota did result in significant immunomodulatory changes in the ileal mucosa and muscularis.

### Metabolic and cellular alterations in the GF colon

**FIGURE 2:**
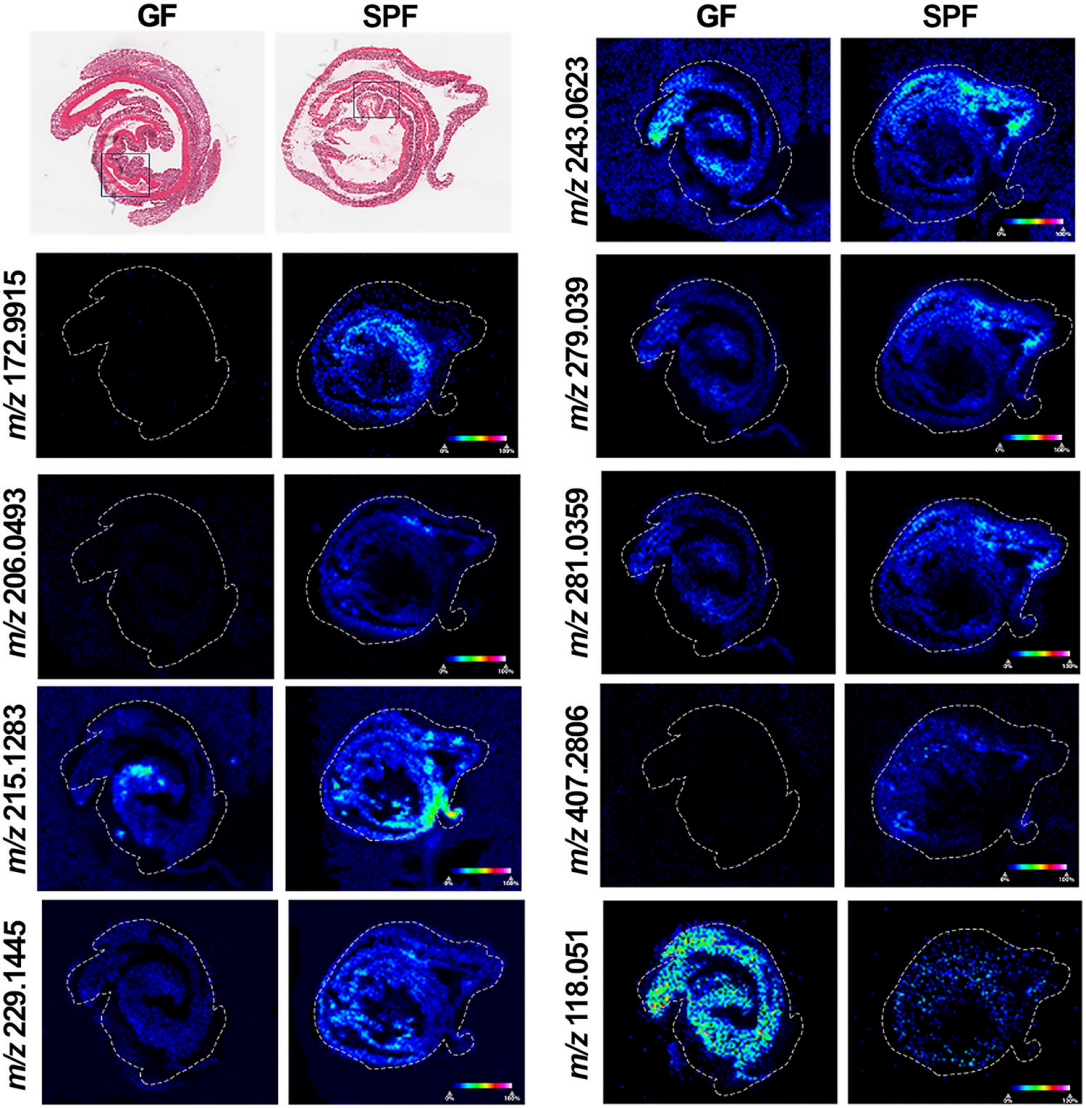
MSI image showing abundance of significantly changed molecules in the colon. From top to bottom: H&E stained sections with rectangular boxes indicating the region selected for IMC, MSI heatmap of *m/z* molecules (color bar shows 0−100% relative abundance). Molecules were putatively identified as phenol sulfate (*m/z* 172.9915), 4‐(2‐aminophenyl)‐2,4‐dioxobutanoic acid (*m/z* 206.0493), unidentified metabolite (*m/z* 215.1283), dodecanedioic acid (*m/z* 229.1445), pseudouridine (*m/z* 243.0623), uridine (*m/z* 279.039), unidentified metabolite (*m/z* 281.0359), cholic acid (*m/z* 407.2806), and l‐threonine (*m/z* 118.051).

ROC analysis post‐MSI identified 17 peaks that could potentially discriminate between the colon in GF and SPF mouse groups. The variation in the 17 metabolites detected was analyzed to examine the clustering of groups using multivariate statistical analysis including PCA and PLS‐DA (Figure S1). PCA and PLS‐DA score plots showed a clear separation between GF and SPF colonic metabolomic profiles and indicated which metabolites were contributing to group separation. The univariate analysis performed indicated that nine of 17 molecules were significantly changed between the groups. The molecules with the following *m/z* were increased in the colon of SPF mice compared to GF mice, and these were putatively identified using the HMDB; phenol sulfate (*m/z* 172.9915, 280.85‐fold, *p* = 0.000373), 4‐(2‐aminophenyl)‐2,4‐dioxobutanoic acid (*m/z* 206.0493, 6.05‐fold, *p* = 0.0062), unidentified metabolite (*m/z* 215.1283, 1.62‐fold, *p* = 0.000929), dodecanedioic acid (DODA) (*m/z* 229.1445, 1.74‐fold, *p* = 0.002363), pseudouridine (*m/z* 243.0623, 1.58‐fold, *p* = 0.041662), uridine (*m/z* 279.039, 2.03‐fold, *p* = 0.007065), unidentified metabolite (*m/z* 281.0359, 2.09‐fold, *p* = 0.008065), and cholic acid (*m/z* 407.2806, 22.42‐fold, *p* = 0.000179) (Figure 2). Lastly, l‐threonine (*m/z* 118.051) was the only molecule decreased in the SPF colon (3.03‐fold; *p* = 0.000061). Pathway analysis focused on these molecules did not identify any specific pathway as enriched in either SPF or GF colons.

**FIGURE 3:**
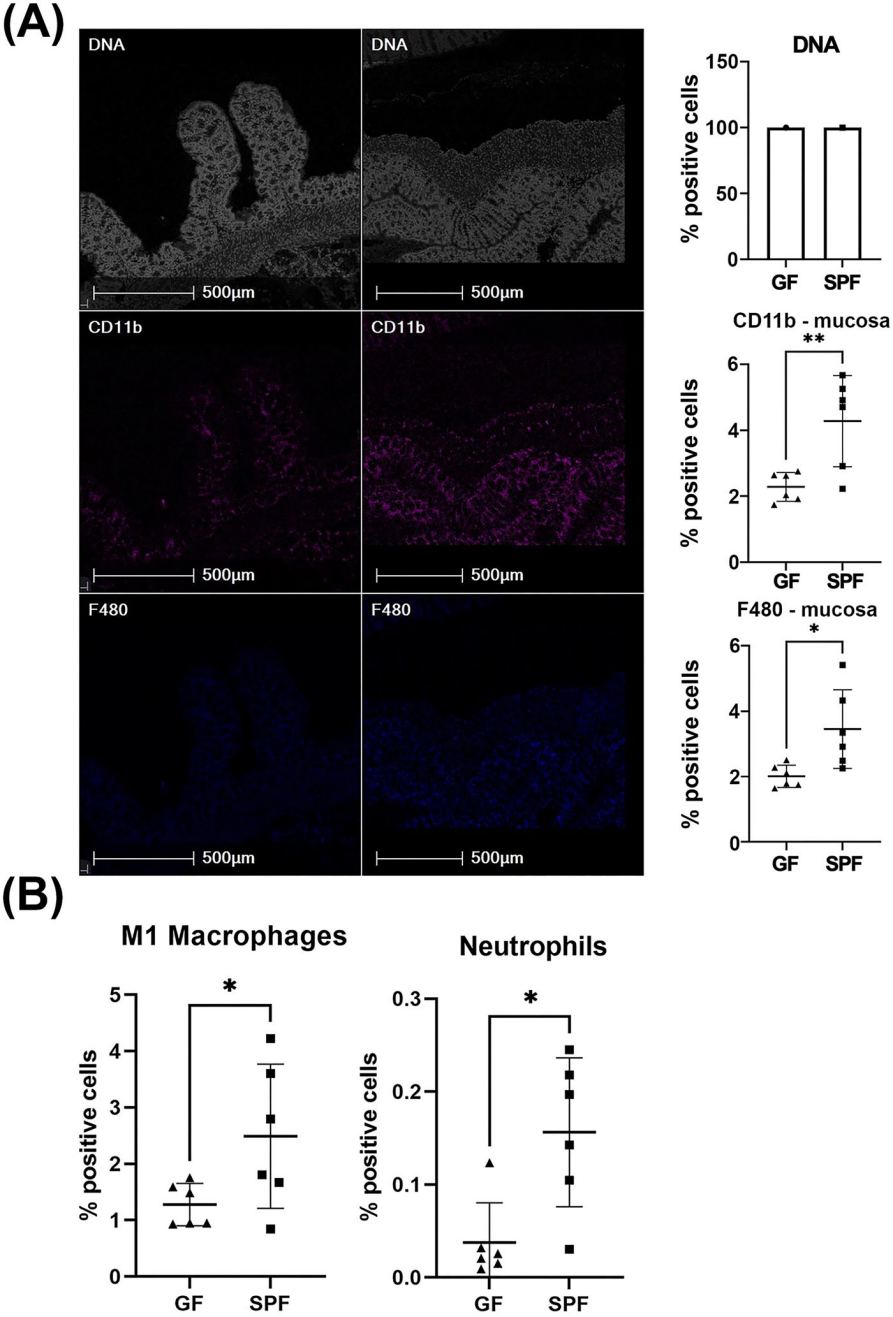
Representative IMC images of biological markers of cell function in the colonic mucosa and muscularis. Images shown are of the region highlighted in the blue rectangle in Figure 2. (A) From top to bottom: DNA intercalator identified single cells within the tissue sections, CD11b and F480. (B) Graph showing the percentage of cells with a phenotype indicative of M1 macrophages and neutrophils. Graphs show mean ± standard error of the mean (SEM) and show four or five biological replicates. For normally distributed data, a two‐tailed *t*‐test was used to determine statistical significance. For neutrophil data, which were not normally distributed, a Mann−Whitney U test was applied. *p<0.05 and **p<0.01 were defined as statistically significant.

IMC was performed on the colon and in the colonic mucosa. CD11b^+^ and F480^+^ expressing cells increased 1.87‐fold (*p* = 0.0071) and 1.71‐fold (*p* = 0.0179), respectively, in SPF compared to GF mice (Figure 3A). The percentage of M1 macrophages (F480^+^, MHCII^+^) and neutrophils (Ly6G^+^, CD11b^+^) were 1.95‐fold (*p* = 0.0496) and 4.15‐fold (*p* = 0.0095) higher, respectively, in the colonic mucosa of SPF mice (Figure 3B). No significantly changed cell markers or phenotypes were found in the colonic muscularis.

### Metabolic alterations in the liver of GF mice

**FIGURE 4:**
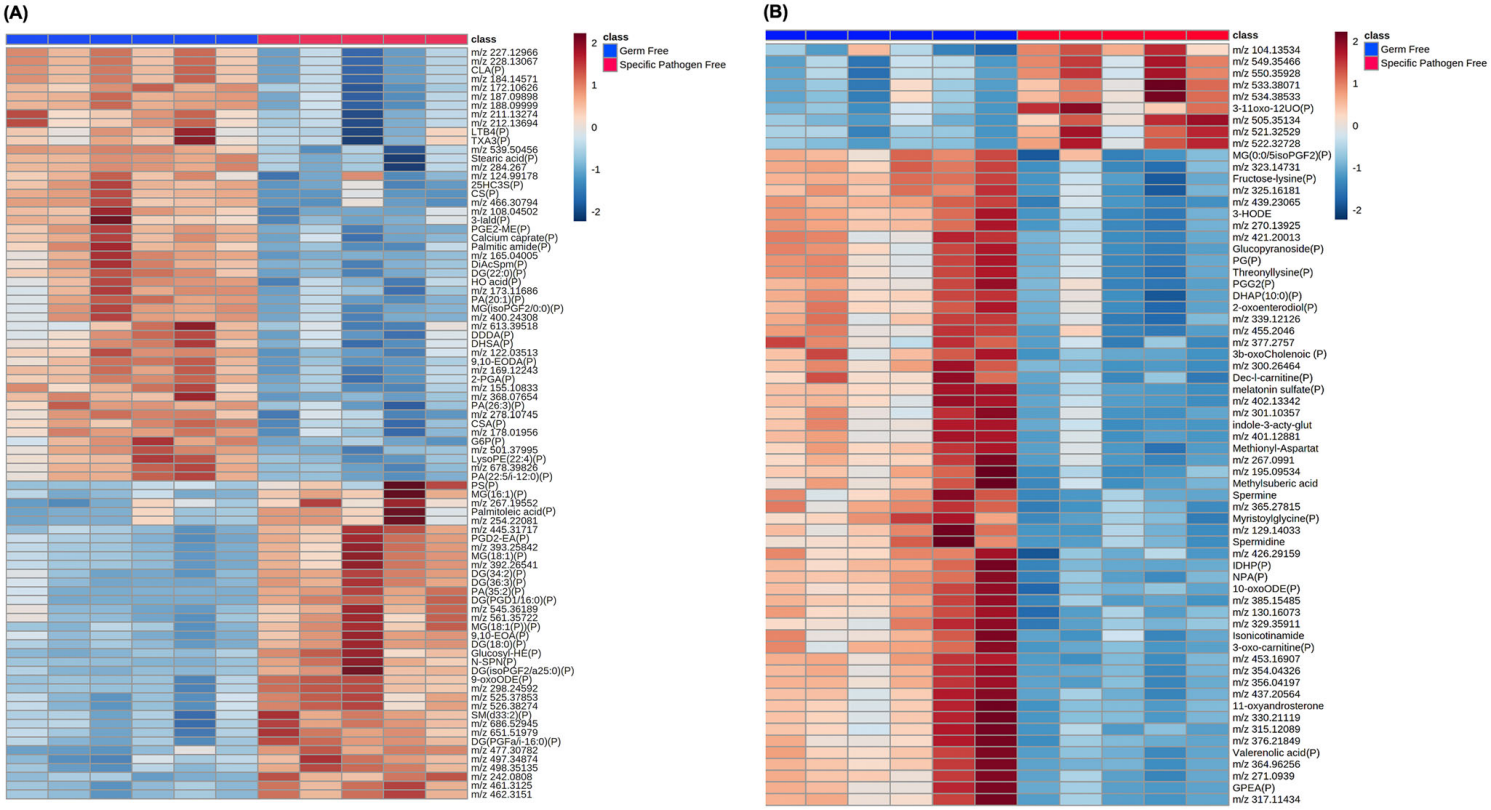
Heatmap of increased and decreased molecules in the liver of germ‐free (GF) and specific pathogen‐free (SPF) mice using negative and positive mode MSI. (A) Heatmap shows *m/z* of molecules detected in (A) negative mode or (B) positive mode. Those unable to be identified are indicated by their *m/z*, molecules with an MSMS‐confirmed identification are named, while those molecules putatively identified are named and marked with a P. The heatmap was created using Metaboanalyst 5.0 and the color bar of each sample is proportional to the significance of change in metabolite abundance and is indicated by the color bar (red, increased; blue, decreased). Rows correspond to metabolites and columns correspond to samples from individual mice (GF, blue; SPF, red).

ROC analysis post‐MSI found 711 peaks that could potentially discriminate between GF and SPF mouse livers. Variation in the 711 molecules detected was analyzed to examine the clustering of groups using multivariate statistical analysis with both PCA and PLS‐DA plots showing clear separation of the groups based on their liver metabolite profile (Figure S2). Molecules with a VIP >1 were selected for univariate analysis, and 250 molecules were found to be significantly changed between GF and SPF livers with 159 of these (63.6%) assigned putative identities using the HMDB. Heatmaps show the molecules detected as increased or decreased in GF and SPF livers using negative mode (Figure 4A) or positive mode DESI‐MSI (Figure 4B). Additionally, Figure S3 highlights the lipids changed in the liver. Of the 250 molecules significantly altered, none were found to overlap with those changed in the ileum, while phenol sulfate (*m/z* 172.9915) was increased in both the colons and livers of SPF mice in comparison to GF mice.

Using the KEGG database, pathway enrichment analysis of molecules significantly altered in the liver indicated that the molecules found are involved in at least 25 pathways. However, only the arachidonic acid (AA) metabolism (*p* = 0.00000125) and amino sugar and nucleotide sugar metabolism (*p* = 0.00189) pathways were significantly enriched. Molecules identified that participate in these pathways are also listed (Figure S4).

### Metabolic and cellular alterations in the lungs of GF mice compared to SPF mice

**FIGURE 5:**
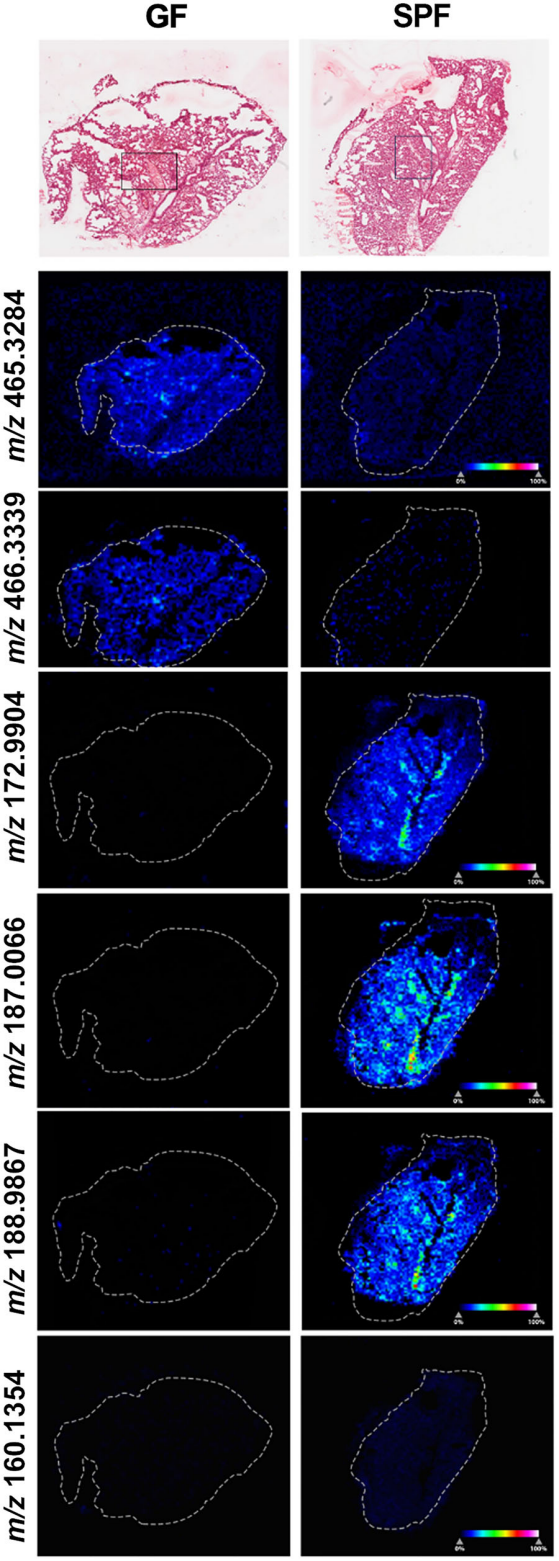
MSI image showing the abundance of significantly changed molecules in the lungs of germ‐free (GF) and specific pathogen‐free (SPF) mice. From top to bottom: H&E stained sections with rectangular boxes indicating the region selected for IMC and MSI heatmap of *m/z* molecules with the color bar showing 0−100% relative abundance.

In the lungs, ROC analysis found only seven peaks that could potentially discriminate between GF and SPF mice. Variation in the seven molecules detected was again analyzed to examine the clustering of groups using multivariate statistical analysis including PLS‐DA and PCA, with score plots from both approaches showing clear separation of the groups (Figure S5). The molecules *m/z* 465.3284 and 466.3339 were found to be significantly decreased by 2.77‐fold (*p* = 0.000019) and 6.15‐fold (*p* = 0.000002), respectively, in the lungs of SPF mice compared to GF mice (Figure 5). The molecule with *m/z* 465.3284 was putatively identified as 3‐hydroxy‐2‐methyl‐6‐{5,9,16‐trihydroxy‐2,15‐dimethyltetracyclo[8.7.0.0^2,7.0^11,15]heptadecan‐14‐yl}heptanoic acid (tetraHCA), whereas *m/z* 466.3339 could not be identified. However, tetraHCA was identified with the adduct M^−^H, and both molecules have similar distribution patterns leading us to conclude that it is likely that *m/z* 466.3339 and *m/z* 465.3284 are the same molecule. Four putatively identified molecules were also significantly increased in the lungs of SPF mice: phenol sulfate (*m/z* 172.9904, 1055.48‐fold, *p* = 0.004204), *p*‐cresol sulfate (*m/z* 187.0066, 1289.82‐fold, *p* = 0.008928), pyrocatechol sulfate (*m/z* 188.9867, 362.95‐fold, *p* = 0.00392), and 5‐AVAB (*m/z* 160.13547, 26.87‐fold, *p* = 0.0035). Due to the low number of molecules identified, pathway enrichment analysis was not possible.

**FIGURE 6:**
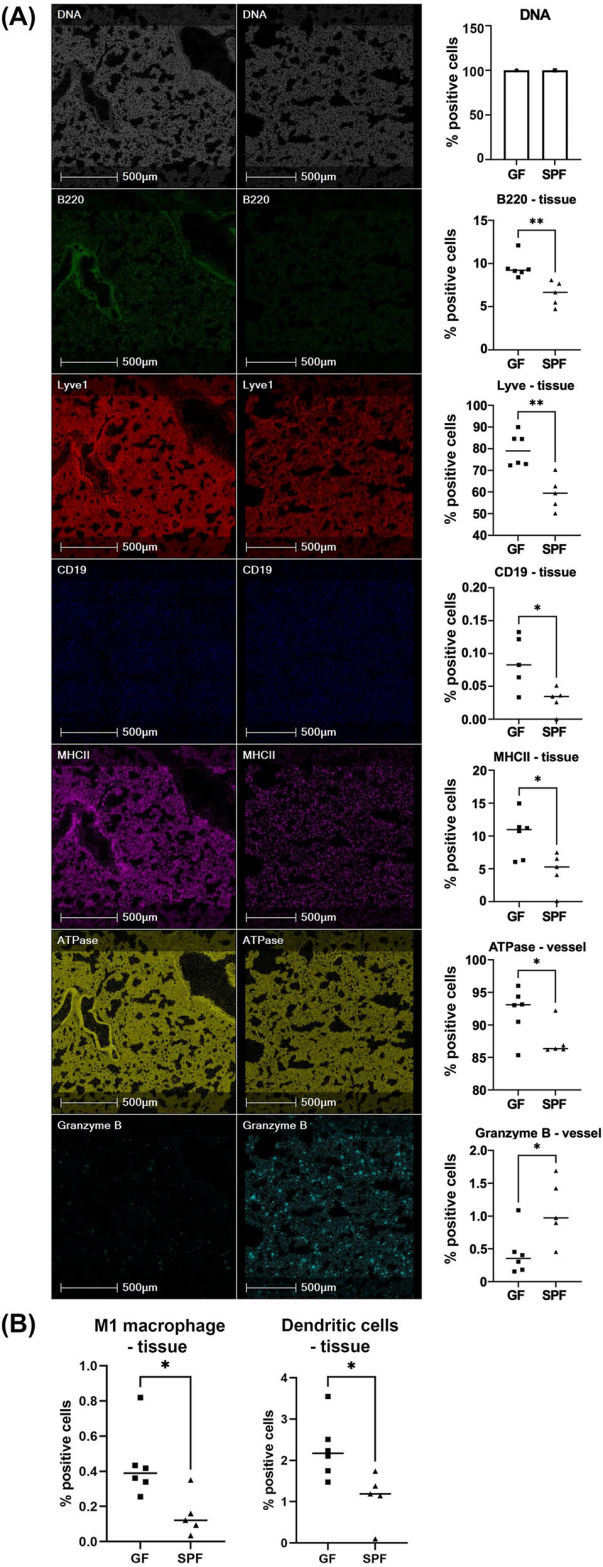
Representative IMC images of biological markers of cell function in the lung tissue and vessels. Images shown are the region indicated in the blue rectangle in Figure 5 for GF and SPF lungs. (A) From top to bottom, DNA intercalator identified single cells within the tissue section, B220, Lyve1, CD19, MHCII, ATPase, and granzyme B. (B) Graph showing the percentage of cells with a phenotype indicative of M1 macrophages and dendritic cells. Graphs show mean ± standard error of the mean (SEM) and show four or five biological replicates. For normally distributed data, a two‐tailed *t*‐test was used to determine statistical significance. For B220 data, which was not normally distributed, a Mann−Whitney U test was applied. *p<0.05 was defined as statistically significant.

Unlike the liver, the lungs exhibited minimal metabolic changes between GF and SPF mice. However, IMC provided a better understanding of immunomodulation that occurred in the absence of substantial metabolomic alterations. Random forest machine learning tissue classifier module segmented the lung into tissue and vessel regions based on morphology and the blood vessel cell marker, CD31. SPF mice had a significant decrease in the percentage of B220^+^ (1.46‐fold; *p* = 0.0043), Lyve1^+^ (1.33‐fold; *p* = 0.0018), CD19^+^ (2.91‐fold; *p* = 0.0294), and MHCII^+^ (2.13‐fold; *p* = 0.02) expressing cells (Figure 6A). The results also indicated that in the SPF lung tissue, the percentage of cells with a phenotype indicative of M1 macrophages and DCs were decreased 2.38‐fold (*p* = 0.0193) and 2.04‐fold (*p* = 0.0194), respectively (Figure 6B). In lung vessels, the percentage of cells expressing ATPase was decreased 1.05‐fold (*p* = 0.05) in SPF compared to GF mice. However, the percentage of cells expressing granzyme B increased 2.51‐fold (*p* = 0.0375) in SPF mice. Therefore, lung immunomodulation appeared dependent on microbiota presence, but independent of local metabolomic changes.

### Metabolic alterations in the spleen and kidneys of GF mice

**FIGURE 7:**
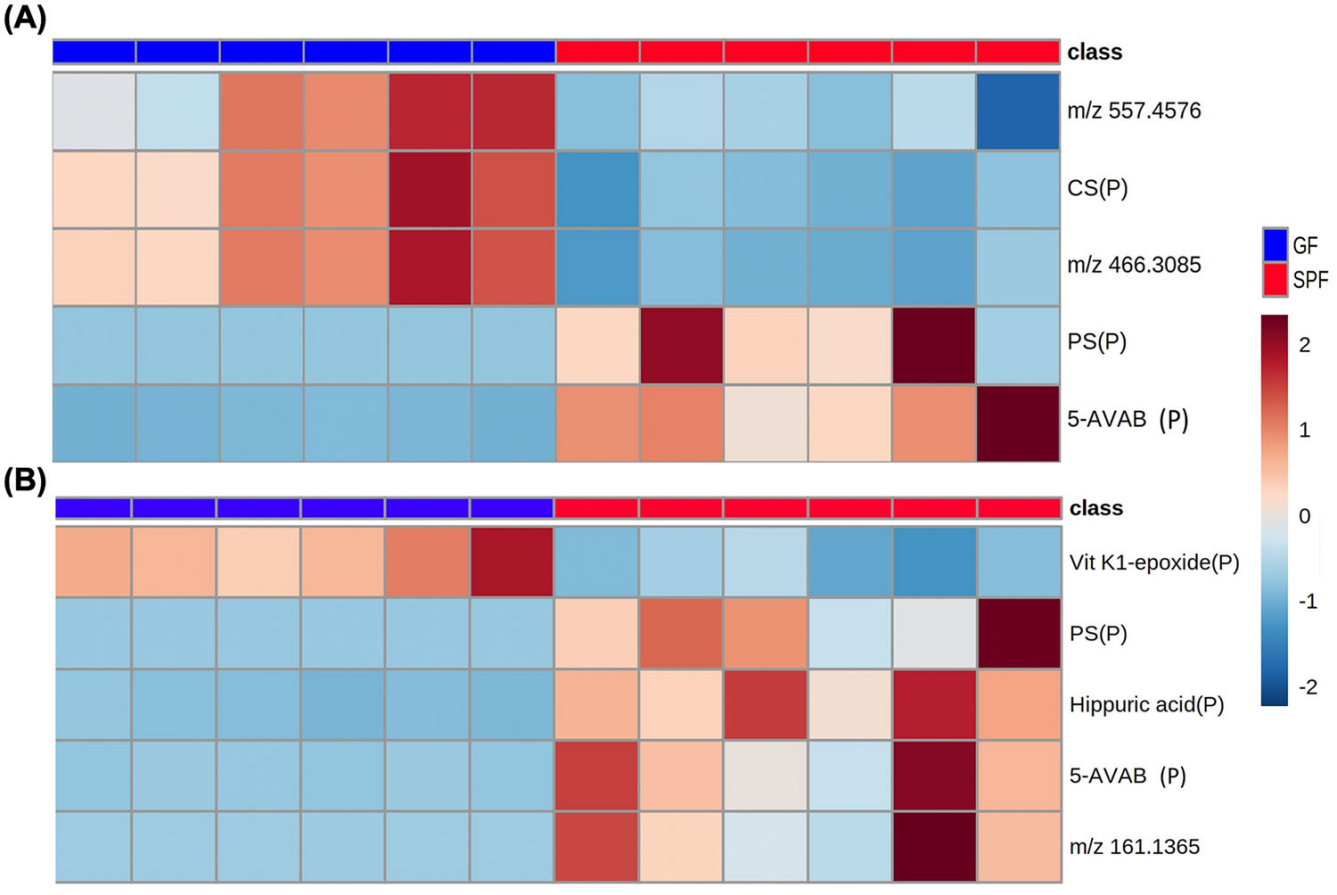
Molecules altered in the spleen and kidneys of germ‐free (GF) and specific pathogen‐free (SPF) mice. Heatmaps show *m/z* of molecules from (A) the spleen or (B) the kidneys unable to be identified and putatively identified molecules (marked with a P). Heatmap was created using Metaboanalyst 5.0. The color of each sample is proportional to the significance of change in metabolite abundance and is indicated by the color bar (red, increased; blue, decreased). Rows correspond to metabolites and columns correspond to samples from individual mice (GF, blue; SPF, red). Abbreviations: CS, *para*‐cresol sulfate; PS, phenol sulfate.

ROC analysis found six peaks that could potentially discriminate between GF and SPF spleens. Variation in the six molecules detected was analyzed to examine the clustering of groups using multivariate statistical analysis including PCA and PLS‐DA (Figure S6). *m/z* 172.9926, putatively identified as phenol sulfate, was increased 988.16‐fold (*p* = 0.0089) in the spleen of SPF mice compared to GF mice (Figure 7A). Furthermore, the molecule *m/z* 160.1318, putatively identified as 5‐AVAB, was increased 25.07‐fold (*p* = 0.0002) in the spleen of SPF mice compared to GF mice. The molecules *m/z* 465.3057 and 466.3084 were significantly decreased in the SPF spleen compared to GF mice by 2.82‐fold (*p* = 0.000035) and 4.03‐fold (*p* = 0.000018), respectively. The molecule with *m/z* 465.3057 was putatively identified as cholesterol sulfate (CS), whereas *m/z* 466.3339 could not be identified. However, CS was identified with the adduct M^−^H, and both molecules have similar distribution patterns meaning it is likely that *m/z* 466.3339 and 465.3284 are the same molecule. Lastly, *m/z* 557.4568 was decreased 1.71‐fold (*p* = 0.0024) in the spleen of SPF mice compared to GF mice. However, the molecule could not be assigned an identity. Pathway enrichment analysis was not possible due to the small number of molecules identified.

The kidney ROC analysis found five peaks that could potentially discriminate between GF and SPF groups. Variation in the five molecules detected was analyzed to examine the clustering of groups using multivariate statistical analysis including PCA and PLS‐DA, and score plots for both PCA and PLS‐DA score showed clear separation in the kidney (Figure S7). The molecule *m/z* 172.9906, putatively identified as phenol sulfate, was increased 2361.54‐fold (*p* = 0.004068) in the kidney of SPF compared to GF mice (Figure 7B). Furthermore, the molecule *m/z* 160.1318, putatively identified as 5‐AVAB, was increased 10.65‐fold (*p* = 0.002913) in the kidney of SPF mice. The molecule *m/z* 178.0512, putatively identified as hippuric acid, was increased 10.03‐fold (*p* = 0.000072) in the kidney of SPF compared to GF mice. Whereas the molecule *m/z* 465.3335, putatively identified as Vitamin K1 2,3‐epoxide, was decreased 2.67‐fold (*p* = 0.000038) in the SPF kidney. As this molecule has a similar *m/z* to the molecule found in the lung (tetraHCA) and spleen (CS), without further analysis, it could not be definitively discounted that they are the same molecule. This study attempted to perform pathway analysis using the putative identities described above but enrichment was again not possible due to the low number of molecules identified.

The only molecules shared between the intestine and systemic tissue were phenol sulfate, which was increased in the SPF colon, liver, lung, spleen, and kidney, and 5‐AVAB, which was increased in the SPF ileum, lung, spleen, and kidney.

## DISCUSSION

To assess whether molecular changes were occurring systemically in response to the presence of a microbiota, MSI was used to investigate molecular changes across a range of organs. Changes were noted in known microbiome‐derived metabolites as well as host metabolites influenced by the microbiome, with the majority of changes detected in host lipid and fatty acid metabolism. *m/z* 160.133, putatively identified as 5‐AVAB, was increased in the ileum, lung, spleen, and kidney of SPF mice compared to GF mice. 5‐AVAB and a number of isomers inhibit fatty acid oxidation and are synthesized by specific members of the gut microbiota, potentially from lysine.^22^, ^23^, ^24^ In contrast, host‐derived DODA, which is increased in the colon of SPF mice, enhances fatty acid oxidation and can enter mitochondria to alleviate energy deficiency and may offer benefits in diseases of mitochondrial dysfunction.^25^, ^26^ Phenol sulfate is another microbiota‐derived gut metabolite that increased systemically in SPF mice.^27^ Increasing levels of microbial phenol sulfate induce reactive oxygen species production, which can result in the progression of diabetes and associated kidney albuminuria in rats.^27^, ^28^ However, while the molecule has been described as a potential biomarker and therapeutic target in diabetic kidney disease, its mechanism of action is not fully understood.^27^, ^29^

In the SPF colon, threonine significantly decreased, while uridine, and its isomer pseudouridine, significantly increased. Threonine deficiency may induce hepatic glyceride accumulation as a protective mechanism to provide the mitochondria with substrates for energy production, something we observed in SPF mice.^30^ Threonine can be degraded by commensal *Clostridium* species, resulting in the production of fatty acids such as acetic acid and butyric acid, which are required for maintaining intestinal barrier function and priming immune responses.^31^ Uridine and pseudouridine play important roles in nucleic acid synthesis and glycosylation making them useful precursors in antitumor and antiviral drugs.^32^, ^33^ However, uremic patients have increased pseudouridine‐producing enzymes in the microbiome, implicating the molecule in kidney disease.^34^

SPF mice had significant changes in lipid metabolism in the liver in comparison to GF mice where diglycerides, triglycerides, ceramides, and sphingomyelins all significantly changed. The AA pathway which produces biologically active molecules such as hydroperoxyeicosatetraenoic acids, prostaglandin, and leukotrienes was also significantly altered.^35^ Prostaglandin G2 which stimulates platelet aggregation and cyclooxygenase activity in inflammatory cells decreased in the liver of SPF mice, suggesting that the gut microbiota exerts an anti‐inflammatory effect.^36^ However, an increase in leukotriene B_4_, another potent lipid mediator that is involved in the recruitment and activation of neutrophils, was also noted in the liver of SPF mice compared to GF mice.^37^ Therefore, the AA pathway in the liver can be modulated by the presence of gut microbiota, and the outcome of this influence may lead to either immunomodulation or disease.

Amino sugar and nucleotide sugar metabolism were significantly enriched in the livers of SPF mice, and molecules such as fructose‐6 phosphate increased.^38^ Nucleotide sugars are building blocks for numerous glycoproteins, glycolipids, and glycosylphosphatidylinositol anchors, and disruption of their synthesis has been implicated in cancer and neurodegenerative conditions.^39^ Furthermore, it has been reported that elevated levels of glucosamine occur in the liver of rats with type 2 diabetes mellitus, while fructose decreases.^40^ This imbalance is thought to contribute to disease onset by inducing bacterial dysbiosis, macrophage activation, and proinflammatory cytokine release as well as reducing insulin sensitivity and again mediating microbiota effects on fatty acid oxidation.^41^

MSMS confirmed the identity of spermidine and spermine in the liver, with both molecules significantly decreased in SPF mice. These naturally occurring polyamines (PAs) are derived from dietary ornithine via decarboxylation and are critical for cellular homeostasis due to their enhancing DNA and RNA stabilization, enzymatic modulation, and regulating translation.^42^ The gut microbiota is a major source of PAs which gain access to the bloodstream via the mucosa. An increase in PAs was, therefore, expected in SPF mice due to their production by the gut microbiota; therefore, the observed reduction indicates that there yet to be unraveled the microbial–host metabolic interactions with respect to PAs.^43^ Spermidine levels decline with aging, and a connection between reduced spermidine concentration and age‐related deterioration has been suggested.^44^ This has been attributed to its role in regulating eukaryotic translation initiation factor 5A and autophagy in the liver, heart, and muscles, resulting in broad health‐promoting effects and longevity.^45^ GF mice exhibit less age‐related inflammation, and on average, live longer than conventional mice, and gut microbiota‐driven decreases in spermidine levels may exert an influence.^46^

Microbially derived phenol sulfate and p‐cresol sulfate (pCS) were detected in the lungs of SPF mice. At least 55 intestinal microbiota species synthesize *p*‐cresol, predominantly in the colon, via conversion of aromatic amino acids such as tyrosine and phenylalanine obtained from the diet.^47^ In the colon and liver, *p*‐cresol is sulfated into *para*‐CS (pCS), which enters circulation, binds plasma albumin, and is filtered in the glomerulus before being excreted in urine.^48^ Patients with chronic kidney disease (CKD) accumulate pCS in the kidneys, which has been associated with worsening outcomes and the development of cardiovascular complications.^49^ Elevated pCS has also been linked to the onset of central nervous system disorders in CKD rodent models, where in the kidney and the brain, it exacerbates cell death, increases oxidative stress, and impairs mitochondrial function leading to vascular disruption.^50^, ^51^, ^52^ In lung tissue, pCS is strongly proinflammatory, promoting alveolar cell death and injury by triggering intracellular reactive oxygen species, release of chemoattractants, and leukocyte activation.^52^, ^53^ Therefore, understanding pCS toxicity, and its effect on different tissue types, might present a new therapeutic strategy for controlling inflammation via microbial modulation.^49^, ^52^, ^53^ At the very least, understanding its systemic effects is critical to understanding the systemic effects of the gut microbiota.

As well as phenol sulfate and 5‐AVAB, CS and *m/z* 466.3084 (the latter not identified), significantly decreased in the spleen of SPF mice. In the kidney, vitamin K1 2,3‐epoxide decreased in SPF mice. Moreover, tetraHCA and *m/z* 466.3339, which again was unidentified, decreased in the lungs of SPF mice. As both CS and tetraHCA were identified with the adduct M^−^H, it is likely that the unidentified molecule with *m/z* 466.3339 is the same molecule plus hydrogen.^54^ As MSI requires a three‐dimensional tissue sample, variations in height can occur leading to inhomogeneity in the acceleration field, resulting in lower mass resolution and mass accuracy.^55^ However, we were unable to confirm the identity of these molecules using MSMS as fragmentation patterns could not be matched with spectra available in online databases. As SPF mice had a decrease in the molecule *m/z* 465.3284 across the spleen, kidney, and lungs, it is possible that it is the same molecule and that MSI limitations have hindered accuracy. TetraHCA belongs to the bile acid class and has been negatively correlated with the severity of atherosclerosis; however, the mechanism underpinning this effect is unknown.^56^ CS can be produced via microbial metabolism of dietary cholesterol and has emerged as an important regulatory molecule for stabilizing cell membranes and supporting platelet adhesion.^57^ Therefore, a decrease in CS may have detrimental effects. Lastly, vitamin K1 2,3‐epoxide is the inactive form of vitamin K, and intestinal bacteria are a major source of vitamin K in the body.^58^ Vitamin K plays an important role in blood coagulation as well as anti‐inflammatory, immunomodulatory, and antitumorigenesis effects in the intestine.

Hippuric acid, which significantly increased in the kidney of SPF mice compared to GF mice, is produced via the conjugation of benzoate with glycine due to the formation of benzoyl‐CoA in the mitochondria of the liver and kidney.^59^ Hippuric acid levels are controlled by the composition of the intestinal microbiota as benzoate is produced in the intestine by the microbial degradation of dietary aromatic compounds. Patients with Crohn's disease have reduced hippuric acid excretion linked to altered gut microbial metabolism of dietary benzoate.^60^ Therefore, it was not surprising that hippuric acid levels are increased in the kidney when a microbiome is present. However, the accumulation of hippuric acid has been positively correlated with the progression of CKD by increasing reactive oxygen species accumulation and promoting the development of renal fibrosis.^61^

The cell proliferation marker Ki67 decreased in the ileum of GF mice, a hallmark of intestinal physiology in GF and microbe‐depleted mice.^62^ Furthermore, F4/80^+^ and CD163^+^ cells, having a phenotype indicative of M2c macrophages, decreased in the ileum of SPF mice compared to GF mice. Macrophages are converted into the anti‐inflammatory M2c subtype after stimulation with IL‐10 before entering apoptosis, and these macrophages limit the duration and intensity of immune and inflammatory reactions in diseases such as acute lung injury and systemic lupus.^63^, ^64^, ^65^ Therefore, the higher levels of M2c macrophages in GF mice may maintain intestinal homeostasis in the absence of a microbe‐primed immune response. As the microbiome has been implicated in the development of inflammatory conditions including inflammatory bowel disease, microbe‐induced immunomodulation involved in human diseases may include the impairment of M2c macrophage function.^63^, ^64^, ^66^

In the ileal muscularis, granzyme B positive cells increased in SPF mice, compared to GF mice. Granzyme B, a serine protease produced by a variety of immune and nonimmune cells, induces inflammation by stimulating cytokine release and extracellular matrix remodeling, including in response to commensal and pathogenic bacteria.^67^, ^68^, ^69^ In addition, phosphorylated serine/threonine‐protein kinase (pAkt^+^)–expressing cells also decreased in the ileal muscularis of SPF mice compared to GF mice. Bifidobacteria decrease the hyperphosphorylation of pAkt in signaling pathways, showing that the gut microbiota can provide a protective effect against oxidative stress and phosphatidylinositide 3‐kinase pathway–associated disease development by reducing pAkt expression.^70^, ^71^

A reduction or absence of microbes and their products is known to significantly decrease neutrophils in neonates and adult mice, and in the current study, this was again seen in the colonic mucosa of GF mice.^72^ This depletion could be rescued following administration of heat‐killed *Escherichia coli* or lipopolysaccharide (LPS), suggesting microbial components mediate neutrophil activation and recruitment.^73^, ^74^ However, there is limited information available detailing the effect of specific metabolites identified in this study on neutrophil function. Therefore, the increase in neutrophils is likely a consequence of gut microbiota presence rather than any one individual metabolite identified here.

Due to the large number of molecular differences in the liver of SPF mice compared to GF mice, it was hypothesized that there would be significant alterations in immune cell phenotypes and function. However, IMC indicated that the only difference in the immune profile was an increase in cells expressing CD45 in SPF mice compared to GF mice. CD45 expression increases during B‐ and T‐cell differentiation and maturation, so the gut microbiota supports the normal function of the immune system. Moreover, studies have shown that the addition of spermidine to mice with psoriasis resulted in a reduction of CD45^+^ immune cells including neutrophils and DC, resulting in dampened autoimmunity.^75^ Therefore, it appears spermidine and CD45 expression are negatively correlated and may be an important mechanism in microbial‐mediated immunomodulation.^45^, ^75^, ^76^ Furthermore, spermidine and spermine have also been shown to exert other anti‐inflammatory and antioxidant properties, enhancing mitochondrial metabolic function, and improving proteostasis.^44^ Studies have also revealed that oral supplementation with spermidine can reduce liver fibrosis and hepatocellular carcinoma by stabilizing the microtubule‐associated protein, MAP1S, to increase autophagy signaling.^77^

In lung tissue, SPF mice had a decrease in the percentage of cells expressing B220 and CD19 mature, resting B cell markers, as well as the APC marker MHCII.^78^, ^79^ Furthermore, the percentage of cells with a phenotype indicative of DCs was reduced in the SPF lung. This suggests that the microenvironment of the SPF lung is immunosuppressive. In patients with chronic obstructive pulmonary disease (COPD), a significant decrease in the number of B cell subsets with a reduced ability to produce anti‐inflammatory IL‐10 worsens the condition.^80^ In contrast, reducing B cells and DCs in the lungs can protect the organ from CD4^+^ T cell–induced inflammation.^80^, ^81^, ^82^ To our knowledge, most GF studies have not reported a difference in B cell and DC number but do report a change in function.^62^ Additionally, granzyme B expression was increased in SPF mice with granzyme B entering the extracellular environment, cleaving extracellular matrix proteins, and contributing to the development of COPD and an emphysematous phenotype in the lungs.^83^ Therefore, the presence of gut microbiota might alter lung function by reducing airflow and energy supply. Further investigation into confirming the identity of specific metabolites in the lungs and assessing their interplay in immunomodulation might highlight new mechanisms for controlling inflammation.

## CONCLUSION

In the current study, we demonstrated that MSI and IMC can be employed to discover metabolic and phenotypic changes in GF mice. Specific microbial metabolites such as 5‐AVAB and phenol sulfate were increased in numerous tissue types including the gut, liver, lungs, spleen, and kidney, and may result in specific outcomes for immunomodulation and host physiology. Multiple metabolites significantly altered by the absence of a microbiota converged on pathways related to fatty acid oxidation and lipid metabolism. Immunological changes were identified in the intestinal mucosa and muscularis that might contribute to organ function, while alterations were also highlighted in the lungs and liver. These could be linked to the presence of microbiota‐associated molecules within the tissue such as spermidine which decreased in the liver of SPF mice and was negatively correlated with CD45 expression. While these findings paint a detailed picture of microbiome metabolites and their effects on the host, they are limited in that they are a snapshot in time and lack temporal resolution. Future studies focusing on fluctuations over time, as well as from multiple animal facilities, will hopefully paint a more detailed picture of the complex interplay between microbe‐derived and host metabolites and their effects on host physiology.

## AUTHOR CONTRIBUTIONS

L.A., R.B., D.M.W., and R.J.A.G. conceived and designed the research; L.A., H.H., O.R., V.T., and C.L. performed the research and acquired the data; L.A., H.H., O.R., C.D., and D.M.W. analyzed and interpreted the data. All authors were involved in drafting and revising the manuscript.

## CONFLICT OF INTEREST STATEMENT

The authors declare that there are no conflicts of interest.

## PEER REVIEW

The peer review history for this article is available at https://publons.com/publon/10.1111/nyas.70002.

## Supporting information



Supplementary Materials.

## Data Availability

The data that support the findings of this study are openly available in the University of Glasgow Petabyte Storage Facility https://doi.org/10.5525/gla.researchdata.1964.

## References

[1] Wernroth, M. L. , Peura, S. , Hedman, A. M. , Hetty, S. , Vicenzi, S. , Kennedy, B. , Fall, K. , Svennblad, B. , Andolf, E. , Pershagen, G. , Theorell‐Haglöw, J. , Nguyen, D. , Sayols‐Baixeras, S. , Dekkers, K. F. , Bertilsson, S. , Almqvist, C. , Dicksved, J. , & Fall, T. (2022). Development of gut microbiota during the first 2 years of life. Scientific Reports, 12(1), 9080. 35641542 10.1038/s41598-022-13009-3 PMC9156670

[2] Belkaid, Y. , & Hand, T. W. (2014). Role of the microbiota in immunity and inflammation. Cell, 157(1), 121–141. 24679531 10.1016/j.cell.2014.03.011 PMC4056765

[3] Roswall, J. , Olsson, L. M. , Kovatcheva‐Datchary, P. , Nilsson, S. , Tremaroli, V. , Simon, M. C. , Kiilerich, P. , Akrami, R. , Krämer, M. , Uhlén, M. , Gummesson, A. , Kristiansen, K. , Dahlgren, J. , & Bäckhed, F. (2021). Developmental trajectory of the healthy human gut microbiota during the first 5 years of life. Cell Host & Microbe, 29(5), 765–776.e3. 33794185 10.1016/j.chom.2021.02.021

[4] Tanaka, M. , & Nakayama, J. (2017). Development of the gut microbiota in infancy and its impact on health in later life. Allergology International, 66(4), 515–522. 28826938 10.1016/j.alit.2017.07.010

[5] Niu, J. , Xu, L. , Qian, Y. , Sun, Z. , Yu, D. , Huang, J. , Zhou, X. , Wang, Y. , Zhang, T. , Ren, R. , Li, Z. , Yu, J. , & Gao, X. (2020). Evolution of the gut microbiome in early childhood: A cross‐sectional study of Chinese children. Frontiers in Microbiology, 11, 439. 32346375 10.3389/fmicb.2020.00439 PMC7169428

[6] Yatsunenko, T. , Rey, F. E. , Manary, M. J. , Trehan, I. , Dominguez‐Bello, M. G. , Contreras, M. , Magris, M. , Hidalgo, G. , Baldassano, R. N. , Anokhin, A. P. , Heath, A. C. , Warner, B. , Reeder, J. , Kuczynski, J. , Caporaso, J. G. , Lozupone, C. A. , Lauber, C. , Clemente, J. C. , Knights, D. , … Gordon, J. I. (2012). Human gut microbiome viewed across age and geography. Nature, 486(7402), 222–227. 22699611 10.1038/nature11053 PMC3376388

[7] Francino, M. P. (2014). Early development of the gut microbiota and immune health. Pathogens, 3(3), 769–790. 25438024 10.3390/pathogens3030769 PMC4243441

[8] Chang, P. V. , Hao, L. , Offermanns, S. , & Medzhitov, R. (2014). The microbial metabolite butyrate regulates intestinal macrophage function via histone deacetylase inhibition. Proceedings of the National Academy of Sciences of the United States of America, 111(6), 2247–2252. 24390544 10.1073/pnas.1322269111 PMC3926023

[9] Wu, K. , Yuan, Y. , Yu, H. , Dai, X. , Wang, S. , Sun, Z. , Wang, F. , Fei, H. , Lin, Q. , Jiang, H. , & Chen, T. (2020). The gut microbial metabolite trimethylamine N‐oxide aggravates GVHD by inducing M1 macrophage polarization in mice. Blood, 136(4), 501–515. 32291445 10.1182/blood.2019003990 PMC7378459

[10] Ivanov, I. I. , Atarashi, K. , Manel, N. , Brodie, E. L. , Shima, T. , Karaoz, U. , Wei, D. , Goldfarb, K. C. , Santee, C. A. , Lynch, S. V. , Tanoue, T. , Imaoka, A. , Itoh, K. , Takeda, K. , Umesaki, Y. , Honda, K. , & Littman, D. R. (2009). Induction of intestinal Th17 cells by segmented filamentous bacteria. Cell, 139(3), 485–498. 19836068 10.1016/j.cell.2009.09.033 PMC2796826

[11] Tan, T. G. , Sefik, E. , Geva‐Zatorsky, N. , Kua, L. , Naskar, D. , Teng, F. , Pasman, L. , Ortiz‐Lopez, A. , Jupp, R. , Wu, H.‐J. J. , Kasper, D. L. , Benoist, C. , & Mathis, D. (2016). Identifying species of symbiont bacteria from the human gut that, alone, can induce intestinal Th17 cells in mice. Proceedings of the National Academy of Sciences of the United States of America, 113(50), E8141–E8150. 27911839 10.1073/pnas.1617460113 PMC5167147

[12] Akdis, M. (2006). Healthy immune response to allergens: T regulatory cells and more. Current Opinion in Immunology, 18(6), 738–744. 17023149 10.1016/j.coi.2006.06.003

[13] Romagnani, S. (2006). Regulation of the T cell response. Clinical and Experimental Allergy, 36(11), 1357–1366. 17083345 10.1111/j.1365-2222.2006.02606.x

[14] Tezuka, H. , & Ohteki, T. (2019). Regulation of IgA production by intestinal dendritic cells and related cells. Frontiers in Immunology, 10, 1891. 31456802 10.3389/fimmu.2019.01891 PMC6700333

[15] Albillos, A. , de Gottardi, A. , & Rescigno, M. (2020). The gut‐liver axis in liver disease: Pathophysiological basis for therapy. Journal of Hepatology, 72(3), 558–577. 31622696 10.1016/j.jhep.2019.10.003

[16] Spadoni, I. , Fornasa, G. , & Rescigno, M. (2017). Organ‐specific protection mediated by cooperation between vascular and epithelial barriers. Nature Reviews Immunology, 17(12), 761–773. 10.1038/nri.2017.100 28869253

[17] Levy, M. , Blacher, E. , & Elinav, E. (2017). Microbiome, metabolites and host immunity. Current Opinion in Microbiology, 35, 8–15. 27883933 10.1016/j.mib.2016.10.003

[18] Han, H. , Wang, M. , Zhong, R. , Yi, B. , Schroyen, M. , & Zhang, H. (2022). Depletion of gut microbiota inhibits hepatic lipid accumulation in high‐fat diet‐fed mice. International Journal of Molecular Sciences, 23(16), 9350. 36012616 10.3390/ijms23169350 PMC9408850

[19] Han, H. , Jiang, Y. I. , Wang, M. , Melaku, M. , Liu, L. , Zhao, Y. , Everaert, N. , Yi, B. , & Zhang, H. (2023). Intestinal dysbiosis in nonalcoholic fatty liver disease (NAFLD): Focusing on the gut‐liver axis. Critical Reviews in Food Science and Nutrition, 63(12), 1689–1706. 34404276 10.1080/10408398.2021.1966738

[20] Adams, L. A. , Wang, Z. , Liddle, C. , Melton, P. E. , Ariff, A. , Chandraratna, H. , Tan, J. , Ching, H. , Coulter, S. , De Boer, B. , Christophersen, C. T. , O'sullivan, T. A. , Morrison, M. , & Jeffrey, G. P. (2020). Bile acids associate with specific gut microbiota, low‐level alcohol consumption and liver fibrosis in patients with non‐alcoholic fatty liver disease. Liver International, 40(6), 1356–1365. 32243703 10.1111/liv.14453

[21] Xia, Y. I. , Ren, M. , Yang, J. , Cai, C. , Cheng, W. , Zhou, X. , Lu, D. , & Ji, F. (2022). Gut microbiome and microbial metabolites in NAFLD and after bariatric surgery: Correlation and causality. Frontiers in Microbiology, 13, 1003755. 36204626 10.3389/fmicb.2022.1003755 PMC9531827

[22] Haikonen, R. , Kärkkäinen, O. , Koistinen, V. , & Hanhineva, K. (2022). Diet‐ and microbiota‐related metabolite, 5‐aminovaleric acid betaine (5‐AVAB), in health and disease. Trends in Endocrinology and Metabolism, 33(7), 463–480. 35508517 10.1016/j.tem.2022.04.004

[23] Hulme, H. , Meikle, L. M. , Strittmatter, N. , Van Der Hooft, J. J. J. , Swales, J. , Bragg, R. A. , Villar, V. H. , Ormsby, M. J. , Barnes, S. , Brown, S. L. , Dexter, A. , Kamat, M. T. , Komen, J. C. , Walker, D. , Milling, S. , Osterweil, E. K. , Macdonald, A. S. , Schofield, C. J. , Tardito, S. , … Wall, D. M. (2020). Microbiome‐derived carnitine mimics as previously unknown mediators of gut‐brain axis communication. Science Advances, 6(11), eaax6328. 32195337 10.1126/sciadv.aax6328 PMC7065903

[24] Zarei, I. , Koistinen, V. M. , Kokla, M. , Klåvus, A. , Babu, A. F. , Lehtonen, M. , Auriola, S. , & Hanhineva, K. (2022). Tissue‐wide metabolomics reveals wide impact of gut microbiota on mice metabolite composition. Scientific Reports, 12(1), 15018. 36056162 10.1038/s41598-022-19327-w PMC9440220

[25] Salinari, S. , Bertuzzi, A. , Gandolfi, A. , Greco, A. V. , Scarfone, A. , Manco, M. , & Mingrone, G. (2006). Dodecanedioic acid overcomes metabolic inflexibility in type 2 diabetic subjects. American Journal of Physiology. Endocrinology and Metabolism, 291(5), E1051–E1058. 16787959 10.1152/ajpendo.00631.2005

[26] Liang, J. , Kou, S. , Chen, C. , Raza, S. H. A. , Wang, S. , Ma, X. , Zhang, W.‐J. , & Nie, C. (2021). Effects of *Clostridium butyricum* on growth performance, metabonomics and intestinal microbial differences of weaned piglets. BMC Microbiology, 21(1), 85. 33752593 10.1186/s12866-021-02143-z PMC7983215

[27] Kikuchi, K. , Saigusa, D. , Kanemitsu, Y. , Matsumoto, Y. , Thanai, P. , Suzuki, N. , Mise, K. , Yamaguchi, H. , Nakamura, T. , Asaji, K. , Mukawa, C. , Tsukamoto, H. , Sato, T. , Oikawa, Y. , Iwasaki, T. , Oe, Y. , Tsukimi, T. , Fukuda, N. N. , Ho, H. J. , … Abe, T. (2019). Gut microbiome‐derived phenyl sulfate contributes to albuminuria in diabetic kidney disease. Nature Communications, 10(1), 1835. 10.1038/s41467-019-09735-4 PMC6478834 31015435

[28] Marhuenda‐Munoz, M. , Laveriano‐Santos, E. P. , Tresserra‐Rimbau, A. , Lamuela‐Raventós, R. M. , Martínez‐Huélamo, M. , & Vallverdú‐Queralt, A. (2019). Microbial phenolic metabolites: Which molecules actually have an effect on human health? Nutrients, 11(11), 2725. 31717653 10.3390/nu11112725 PMC6893422

[29] Kikuchi, K. , Itoh, Y. , Tateoka, R. , Ezawa, A. , Murakami, K. , & Niwa, T. (2010). Metabolomic analysis of uremic toxins by liquid chromatography/electrospray ionization‐tandem mass spectrometry. Journal of Chromatography. B, Analytical Technologies in the Biomedical and Life Sciences, 878(20), 1662–1668. 20036201 10.1016/j.jchromb.2009.11.040

[30] Ross‐Inta, C. M. , Zhang, Y.‐F. , Almendares, A. , & Giulivi, C. (2009). Threonine‐deficient diets induced changes in hepatic bioenergetics. American Journal of Physiology Gastrointestinal and Liver Physiology, 296(5), G1130–G1139. 19228885 10.1152/ajpgi.90545.2008 PMC2696218

[31] Neis, E. P. , Dejong, C. H. , & Rensen, S. S. (2015). The role of microbial amino acid metabolism in host metabolism. Nutrients, 7(4), 2930–2946. 25894657 10.3390/nu7042930 PMC4425181

[32] Morais, P. , Adachi, H. , & Yu, Y. T. (2021). The critical contribution of pseudouridine to mRNA COVID‐19 vaccines. Frontiers in Cell and Developmental Biology, 9, 789427. 34805188 10.3389/fcell.2021.789427 PMC8600071

[33] Cheng, K.‐G. , Su, C. H. , Huang, J. Y. , Liu, J. , Zheng, Y. T. , & Chen, Z. F. (2016). Conjugation of uridine with oleanolic acid derivatives as potential antitumor agents. Chemical Biology and Drug Design, 88(3), 329–340. 26990000 10.1111/cbdd.12758

[34] Popkov, V. A. , Zharikova, A. A. , Demchenko, E. A. , Andrianova, N. V. , Zorov, D. B. , & Plotnikov, E. Y. (2022). Gut microbiota as a source of uremic toxins. International Journal of Molecular Sciences, 23(1), 483. 35008909 10.3390/ijms23010483 PMC8745165

[35] Wang, B. , Wu, L. , Chen, J. , Dong, L. , Chen, C. , Wen, Z. , Hu, J. , Fleming, I. , & Wang, D. W. (2021). Metabolism pathways of arachidonic acids: Mechanisms and potential therapeutic targets. Signal Transduction and Targeted Therapy, 6(1), 94. 33637672 10.1038/s41392-020-00443-w PMC7910446

[36] Kuehl, F. A. , Humes, J. L. , Egan, R. W. , Ham, E. A. , Beveridge, G. C. , & Arman, C. G. V. (1977). Role of prostaglandin endoperoxide PGG2 in inflammatory processes. Nature, 265(5590), 170–173. 834259 10.1038/265170a0

[37] Asahara, M. , Ito, N. , Hoshino, Y. , Sasaki, T. , Yokomizo, T. , Nakamura, M. , Shimizu, T. , & Yamada, Y. (2022). Role of leukotriene B4 (LTB4)‐LTB4 receptor 1 signaling in post‐incisional nociceptive sensitization and local inflammation in mice. PLoS ONE, 17(10), e0276135. 36264904 10.1371/journal.pone.0276135 PMC9584502

[38] van Scherpenzeel, M. , Conte, F. , Büll, C. , Ashikov, A. , Hermans, E. , Willems, A. , van Tol, W. , Kragt, E. , Noga, M. , Moret, E. E. , Heise, T. , Langereis, J. D. , Rossing, E. , Zimmermann, M. , Rubio‐Gozalbo, M. E. , de Jonge, M. I. , Adema, G. J. , Zamboni, N. , Boltje, T. , & Lefeber, D. J. (2022). Dynamic tracing of sugar metabolism reveals the mechanisms of action of synthetic sugar analogs. Glycobiology, 32(3), 239–250. 34939087 10.1093/glycob/cwab106 PMC8966471

[39] Moriwaki, K. , Noda, K. , Furukawa, Y. , Ohshima, K. , Uchiyama, A. , Nakagawa, T. , Taniguchi, N. , Daigo, Y. , Nakamura, Y. , Hayashi, N. , & Miyoshi, E. (2009). Deficiency of GMDS leads to escape from NK cell‐mediated tumor surveillance through modulation of TRAIL signaling. Gastroenterology, 137(1), 188–198. e2198 e1‐2. 19361506 10.1053/j.gastro.2009.04.002

[40] Yu, Y. , Lu, Q. , Chen, F. , Wang, S. , Niu, C. , Liao, J. , Wang, H. , & Chen, F. (2022). Serum untargeted metabolomics analysis of the mechanisms of evodiamine on type 2 diabetes mellitus model rats. Food & Function, 13(12), 6623–6635. 35635367 10.1039/d1fo04396j

[41] He, R. , Li, Y. , Han, C. , Lin, R. , Qian, W. , & Hou, X. (2019). L‐Fucose ameliorates DSS‐induced acute colitis via inhibiting macrophage M1 polarization and inhibiting NLRP3 inflammasome and NF‐kB activation. International Immunopharmacology, 73, 379–388. 31132733 10.1016/j.intimp.2019.05.013

[42] Pucciarelli, S. , Moreschini, B. , Micozzi, D. , De Fronzo, G. S. , Carpi, F. M. , Polzonetti, V. , Vincenzetti, S. , Mignini, F. , & Napolioni, V. (2012). Spermidine and spermine are enriched in whole blood of nona/centenarians. Rejuvenation Research, 15(6), 590–595. 22950434 10.1089/rej.2012.1349

[43] Tofalo, R. , Cocchi, S. , & Suzzi, G. (2019). Polyamines and gut microbiota. Frontiers in Nutrition, 6, 16. 30859104 10.3389/fnut.2019.00016 PMC6397830

[44] Madeo, F. , Bauer, M. A. , Carmona‐Gutierrez, D. , & Kroemer, G. (2019). Spermidine: A physiological autophagy inducer acting as an anti‐aging vitamin in humans? Autophagy, 15(1), 165–168. 30306826 10.1080/15548627.2018.1530929 PMC6287690

[45] Eisenberg, T. , Knauer, H. , Schauer, A. , Büttner, S. , Ruckenstuhl, C. , Carmona‐Gutierrez, D. , Ring, J. , Schroeder, S. , Magnes, C. , Antonacci, L. , Fussi, H. , Deszcz, L. , Hartl, R. , Schraml, E. , Criollo, A. , Megalou, E. , Weiskopf, D. , Laun, P. , Heeren, G. , … Madeo, F. (2009). Induction of autophagy by spermidine promotes longevity. Nature Cell Biology, 11(11), 1305–1314. 19801973 10.1038/ncb1975

[46] Fransen, F. , Van Beek, A. A. , Borghuis, T. , Aidy, S. E. L. , Hugenholtz, F. , Van Der Gaast‐De Jongh, C. , Savelkoul, H. F. J. , De Jonge, M. I. , Boekschoten, M. V. , Smidt, H. , Faas, M. M. , & De Vos, P. (2017). Aged gut microbiota contributes to systemical inflammaging after transfer to germ‐free mice. Frontiers in Immunology, 8, 1385. 29163474 10.3389/fimmu.2017.01385 PMC5674680

[47] Bermudez‐Martin, P. , Becker, J. A. J. , Caramello, N. , Fernandez, S. P. , Costa‐Campos, R. , Canaguier, J. , Barbosa, S. , Martinez‐Gili, L. , Myridakis, A. , Dumas, M. E. , Bruneau, A. , Cherbuy, C. , Langella, P. , Callebert, J. , Launay, J. M. , Chabry, J. , Barik, J. , Le Merrer, J. , Glaichenhaus, N. , & Davidovic, L. (2021). The microbial metabolite p‐Cresol induces autistic‐like behaviors in mice by remodeling the gut microbiota. Microbiome, 9(1), 157. 34238386 10.1186/s40168-021-01103-z PMC8268286

[48] Meyer, T. W. , & Hostetter, T. H. (2012). Uremic solutes from colon microbes. Kidney International, 81(10), 949–954. 22318422 10.1038/ki.2011.504

[49] Bao, M. , Zhang, P. , Guo, S. , Zou, J. , Ji, J. , Ding, X. , & Yu, X. (2022). Altered gut microbiota and gut‐derived p‐cresyl sulfate serum levels in peritoneal dialysis patients. Frontiers in Cellular and Infection Microbiology, 12, 639624. 36237423 10.3389/fcimb.2022.639624 PMC9551184

[50] Sun, C. Y. , Li, J.‐R. , Wang, Y.‐Y. , Lin, S.‐Y. , Ou, Y. C. , Lin, C. J. , Wang, J. D. , Liao, S.‐L. , & Chen, C. J. (2020). p‐Cresol sulfate caused behavior disorders and neurodegeneration in mice with unilateral nephrectomy involving oxidative stress and neuroinflammation. International Journal of Molecular Sciences, 21(18), 6687. 32932690 10.3390/ijms21186687 PMC7555291

[51] Liu, W. C. , Tomino, Y. , & Lu, K. C. (2018). Impacts of indoxyl sulfate and p‐cresol sulfate on chronic kidney disease and mitigating effects of AST‐120. Toxins (Basel), 10(9), 367. 30208594 10.3390/toxins10090367 PMC6162782

[52] Chang, J. F. , Liang, S. S. , Thanasekaran, P. , Chang, H. W. , Wen, L.‐L. , Chen, C. H. , Liou, J. C. , Yeh, J. C. , Liu, S. H. , Dai, H. M. , & Lin, W. N. (2018). Translational medicine in pulmonary‐renal crosstalk: Therapeutic targeting of p‐cresyl sulfate triggered nonspecific ROS and chemoattractants in dyspneic patients with uremic lung injury. Journal of Clinical Medicine, 7(9), 266. 30205620 10.3390/jcm7090266 PMC6162871

[53] Campillo, S. , Bohorquez, L. , Gutiérrez‐Calabrés, E. , García‐Ayuso, D. , Miguel, V. , Griera, M. , Calle, Y. , De Frutos, S. , Rodríguez‐Puyol, M. , Rodríguez‐Puyol, D. , & Calleros, L. (2022). Indoxyl sulfate‐ and P‐cresol‐induced monocyte adhesion and migration is mediated by integrin‐linked kinase‐dependent podosome formation. Experimental & Molecular Medicine, 54(3), 226–238. 35246616 10.1038/s12276-022-00738-8 PMC8980039

[54] Zhu, J. , & Cole, R. B. (2000). Formation and decompositions of chloride adduct ions. Journal of the American Society for Mass Spectrometry, 11(11), 932–941. 11073256 10.1016/s1044-0305(00)00164-1

[55] Römpp, A. , & Spengler, B. (2013). Mass spectrometry imaging with high resolution in mass and space. Histochemistry and Cell Biology, 139(6), 759–783. 23652571 10.1007/s00418-013-1097-6 PMC3656243

[56] Hu, X. , Fan, Y. , Li, H. , Zhou, R. , Zhao, X. , Sun, Y. , & Zhang, S. (2021). Impacts of cigarette smoking status on metabolomic and gut microbiota profile in male patients with coronary artery disease: A multi‐omics study. Frontiers in Cardiovascular Medicine, 8, 766739. 34778417 10.3389/fcvm.2021.766739 PMC8581230

[57] Hanyu, O. , Nakae, H. , Miida, T. , Higashi, Y. , Fuda, H. , Endo, M. , Kohjitani, A. , Sone, H. , & Strott, C. A. (2012). Cholesterol sulfate induces expression of the skin barrier protein filaggrin in normal human epidermal keratinocytes through induction of RORalpha. Biochemical and Biophysical Research Communications, 428(1), 99–104. 23063684 10.1016/j.bbrc.2012.10.013

[58] Yan, H. , Chen, Y. , Zhu, H. , Huang, W. H. , Cai, X.‐H. , Li, D. , Lv, Y.‐J. , Si‐Zhao , Zhou, H. H. , Luo, F. Y. , Zhang, W. , & Li, X. (2022). The relationship among intestinal bacteria, vitamin K and response of vitamin K antagonist: A review of evidence and potential mechanism. Frontiers in Medicine (Lausanne), 9, 829304. 10.3389/fmed.2022.829304 PMC9058076 35510250

[59] Caldwell, J. , Moffatt, J. R. , & Smith, R. L. (1976). Post‐mortem survival of hippuric acid formation in rat and human cadaver tissue samples. Xenobiotica, 6(5), 275–280. 936647 10.3109/00498257609151639

[60] Williams, H. R. , Cox, I. J. , Walker, D. G. , Cobbold, J. F. , Taylor‐Robinson, S. D. , Marshall, S. E. , & Orchard, T. R. (2010). Differences in gut microbial metabolism are responsible for reduced hippurate synthesis in Crohn's disease. BMC Gastroenterology [Electronic Resource], 10, 108. 20849615 10.1186/1471-230X-10-108 PMC2954941

[61] Sun, B. , Wang, X. , Liu, X. , Wang, L. , Ren, F. , Wang, X. , & Leng, X. (2020). Hippuric acid promotes renal fibrosis by disrupting redox homeostasis via facilitation of NRF2‐KEAP1‐CUL3 interactions in chronic kidney disease. Antioxidants (Basel), 9(9), 783. 32854194 10.3390/antiox9090783 PMC7555723

[62] Kennedy, E. A. , King, K. Y. , & Baldridge, M. T. (2018). Mouse microbiota models: Comparing germ‐free mice and antibiotics treatment as tools for modifying gut bacteria. Frontiers in Physiology, 9, 1534. 30429801 10.3389/fphys.2018.01534 PMC6220354

[63] Rojas, J. , Salazar, J. , Martínez, M. S. , Palmar, J. , Bautista, J. , Chávez‐Castillo, M. , Gómez, A. , & Bermúdez, V. (2015). Macrophage heterogeneity and plasticity: Impact of macrophage biomarkers on atherosclerosis. Scientifica (Cairo), 2015, 851252. 26491604 10.1155/2015/851252 PMC4600540

[64] Hilliard, B. A. , Zizzo, G. , Ulas, M. , Linan, M. K. , Schreiter, J. , & Cohen, P. L. (2014). Increased expression of Mer tyrosine kinase in circulating dendritic cells and monocytes of lupus patients: Correlations with plasma interferon activity and steroid therapy. Arthritis Research & Therapy, 16(2), R76. 24650765 10.1186/ar4517 PMC4060208

[65] Bijarchian, F. , Taghiyar, L. , Azhdari, Z. , & Baghaban Eslaminejad, M. (2021). M2c macrophages enhance phalange regeneration of amputated mice digits in an organ co‐culture system. Iranian Journal of Basic Medical Sciences, 24(11), 1602–1612. 35317116 10.22038/IJBMS.2021.57887.12870 PMC8917845

[66] Yang, R. , Liao, Y. , Wang, L. , He, P. , Hu, Y. , Yuan, D. , Wu, Z. , & Sun, X. (2019). Exosomes derived from M2b macrophages attenuate DSS‐induced colitis. Frontiers in Immunology, 10, 2346. 31749791 10.3389/fimmu.2019.02346 PMC6843072

[67] Velotti, F. , Barchetta, I. , Cimini, F. A. , & Cavallo, M. G. (2020). Granzyme B in inflammatory diseases: Apoptosis, inflammation, extracellular matrix remodeling, epithelial‐to‐mesenchymal transition and fibrosis. Frontiers in Immunology, 11, 587581. 33262766 10.3389/fimmu.2020.587581 PMC7686573

[68] Trapani, J. A. , & Sutton, V. R. (2003). Granzyme B: Pro‐apoptotic, antiviral and antitumor functions. Current Opinion in Immunology, 15(5), 533–543. 14499262 10.1016/s0952-7915(03)00107-9

[69] Castleman, M. J. , Dillon, S. M. , Thompson, T. A. , Santiago, M. L. , Mccarter, M. D. , Barker, E. , & Wilson, C. C. (2021). Gut bacteria induce granzyme B expression in human colonic ILC3s in vitro in an IL‐15‐dependent manner. Journal of Immunology, 206(12), 3043–3052. 10.4049/jimmunol.2000239 PMC8664910 34117105

[70] Oroojzadeh, P. , Bostanabad, S. Y. , & Lotfi, H. (2022). Psychobiotics: The influence of gut microbiota on the gut‐brain axis in neurological disorders. Journal of Molecular Neuroscience, 72(9), 1952–1964. 35849305 10.1007/s12031-022-02053-3 PMC9289355

[71] Bonfili, L. , Cecarini, V. , Gogoi, O. , Berardi, S. , Scarpona, S. , Angeletti, M. , Rossi, G. , & Eleuteri, A. M. (2020). Gut microbiota manipulation through probiotics oral administration restores glucose homeostasis in a mouse model of Alzheimer's disease. Neurobiology of Aging, 87, 35–43. 31813629 10.1016/j.neurobiolaging.2019.11.004

[72] Zhang, D. , & Frenette, P. S. (2019). Cross talk between neutrophils and the microbiota. Blood, 133(20), 2168–2177. 30898860 10.1182/blood-2018-11-844555 PMC6524562

[73] Deshmukh, H. S. , Liu, Y. , Menkiti, O. R. , Mei, J. , Dai, N. , O'leary, C. E. , Oliver, P. M. , Kolls, J. K. , Weiser, J. N. , & Worthen, G. S. (2014). The microbiota regulates neutrophil homeostasis and host resistance to *Escherichia coli* K1 sepsis in neonatal mice. Nature Medicine, 20(5), 524–530. 10.1038/nm.3542 PMC4016187 24747744

[74] Khosravi, A. , Yáñez, A. , Price, J. G. , Chow, A. , Merad, M. , Goodridge, H. S. , & Mazmanian, S. K. (2014). Gut microbiota promote hematopoiesis to control bacterial infection. Cell Host & Microbe, 15(3), 374–381. 24629343 10.1016/j.chom.2014.02.006 PMC4144825

[75] Li, G. , Ding, H. , Yu, X. , Meng, Y. , Li, J. , Guo, Q. , Zhou, H. , & Shen, N. (2020). Spermidine suppresses inflammatory DC function by activating the FOXO3 pathway and counteracts autoimmunity. Iscience, 23(1), 100807. 31962236 10.1016/j.isci.2019.100807 PMC6971394

[76] Hofer, S. J. , Simon, A. K. , Bergmann, M. , Eisenberg, T. , Kroemer, G. , & Madeo, F. (2022). Mechanisms of spermidine‐induced autophagy and geroprotection. Nature Aging, 2(12), 1112–1129. 37118547 10.1038/s43587-022-00322-9

[77] Yue, F. , Li, W. , Zou, J. , Jiang, X. , Xu, G. , Huang, H. , & Liu, L. (2017). Spermidine prolongs lifespan and prevents liver fibrosis and hepatocellular carcinoma by activating MAP1S‐mediated autophagy. Cancer Research, 77(11), 2938–2951. 28386016 10.1158/0008-5472.CAN-16-3462 PMC5489339

[78] Rodig, S. J. , Shahsafaei, A. , Li, B. , & Dorfman, D. M. (2005). The CD45 isoform B220 identifies select subsets of human B cells and B‐cell lymphoproliferative disorders. Human Pathology, 36(1), 51–57. 15712182 10.1016/j.humpath.2004.10.016

[79] Wosen, J. E. , Mukhopadhyay, D. , Macaubas, C. , & Mellins, E. D. (2018). Epithelial MHC class II expression and its role in antigen presentation in the gastrointestinal and respiratory tracts. Frontiers in Immunology, 9, 2144. 30319613 10.3389/fimmu.2018.02144 PMC6167424

[80] Courtemanche, O. , Huppé, C. A. , Blais Lecours, P. , Lerdu, O. , Roy, J. , Lauzon‐Joset, J. F. , Blanchet, M. R. , Morissette, M. C. , & Marsolais, D. (2022). Co‐modulation of T cells and B cells enhances the inhibition of inflammation in experimental hypersensitivity pneumonitis. Respiratory Research, 23(1), 275. 36209215 10.1186/s12931-022-02200-9 PMC9547367

[81] Jacobs, M. , Verschraegen, S. , Salhi, B. , Anckaert, J. , Mestdagh, P. , Brusselle, G. G. , & Bracke, K. R. (2022). IL‐10 producing regulatory B cells are decreased in blood from smokers and COPD patients. Respiratory Research, 23(1), 287. 36253785 10.1186/s12931-022-02208-1 PMC9578234

[82] Chougnet, C. A. , Thacker, R. I. , Shehata, H. M. , Hennies, C. M. , Lehn, M. A. , Lages, C. S. , & Janssen, E. M. (2015). Loss of phagocytic and antigen cross‐presenting capacity in aging dendritic cells is associated with mitochondrial dysfunction. Journal of Immunology, 195(6), 2624–2632. 10.4049/jimmunol.1501006 PMC4561185 26246142

[83] Ngan, D. A. , Vickerman, S. V. , Granville, D. J. , Man, S. F. P. , & Sin, D. D. (2009). The possible role of granzyme B in the pathogenesis of chronic obstructive pulmonary disease. Therapeutic Advances in Respiratory Disease, 3(3), 113–129. 19638369 10.1177/1753465809341965

