# Supplementary material for: Spatial metabolic and phenotypic characterization of the germ‐free mouse model
Source: Ann N Y Acad Sci. 2025 Aug 4;1551(1):242–56. doi: 10.1111/nyas.70002 (PMC12448274; doi:10.1111/nyas.70002)
Supplement: Supplementary file 1 — Supplementary Materials. [file NYAS-1551-242-s001.docx]

**Spatial metabolic and phenotypic characterisation of the germ-free mouse model**

Lauren Adams^1^, Heather Hulme^2^, Clio Dritsa^1^, Connor Lynch^3^, Vicky Taylor^3^, Orhan Rasid^1^, Richard Burchmore^1^, Richard J.A. Goodwin^1,2^, Daniel M. Wall^1^

^1^School of Infection and Immunology, College of Medical, Veterinary and Life Sciences, Sir Graeme Davies Building, University of Glasgow, Glasgow G12 8TA, United Kingdom.

^2^Integrated Bioanalysis, Clinical Pharmacology and Safety Sciences, BioPharmaceuticals R&D, AstraZeneca, Cambridge, CB4 0WG, United Kingdom.

^3^Biological Services Facility, The University of Manchester, Manchester, M13 9PL, United Kingdom.

*Corresponding author email address:* [[Donal.Wall@glasgow.ac.uk](mailto:Donal.Wall@glasgow.ac.ukD)](mailto:Donal.Wall@glasgow.ac.uk)

Keywords:

Spatial biology, microbiome, imaging, germ-free, molecules

**Supplementary Materials**

**Supplementary Materials**

**Supplementary Figures**


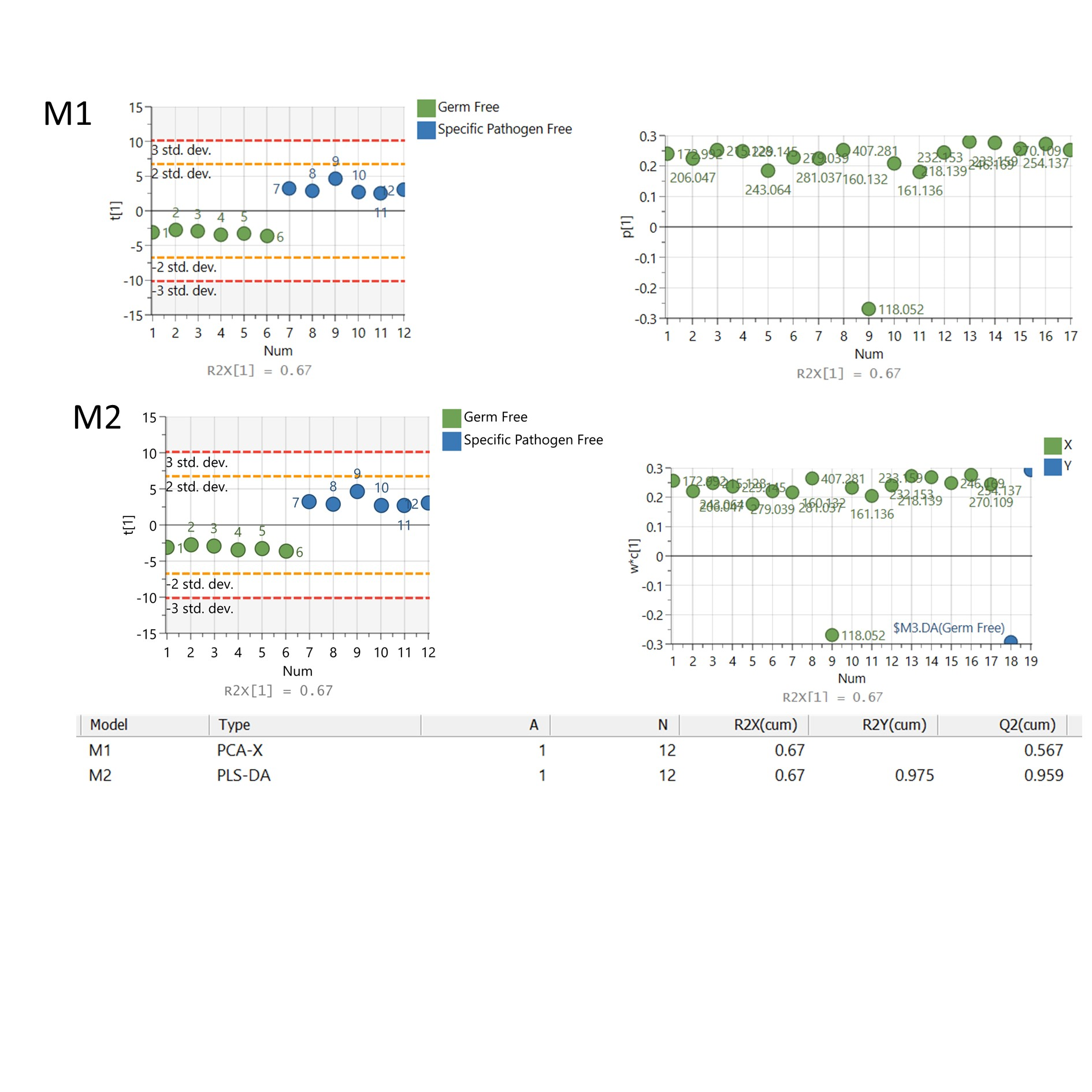


**Figure S1: Unsupervised and supervised discriminant analysis of metabolites found in the colon.** M1) Unsupervised PCA analysis was able to discriminate between the groups using metabolite features found in the colon. M2) Supervised PLS-DA analysis also shows that the molecules in the colon can discriminate between groups (GF, green circles; SPF, blue circles). PCA score plots of the first (t [1]) principal component did show clear separation in the colon between GF and SPF mice (parameters: R2X=0.67, Q2=0.563). PLS-DA score plot (M2) was also able to separate the groups based on the colonic metabolomic profile (parameters: R2X=0.67, R2Y=0.975, Q2=0.959). Corresponding loadings plots indicate which metabolites might contribute to group separation. Analysis was performed using SIMCA 17 software. PLS-DA and PCA score plots consist of component 1 (t [1]).


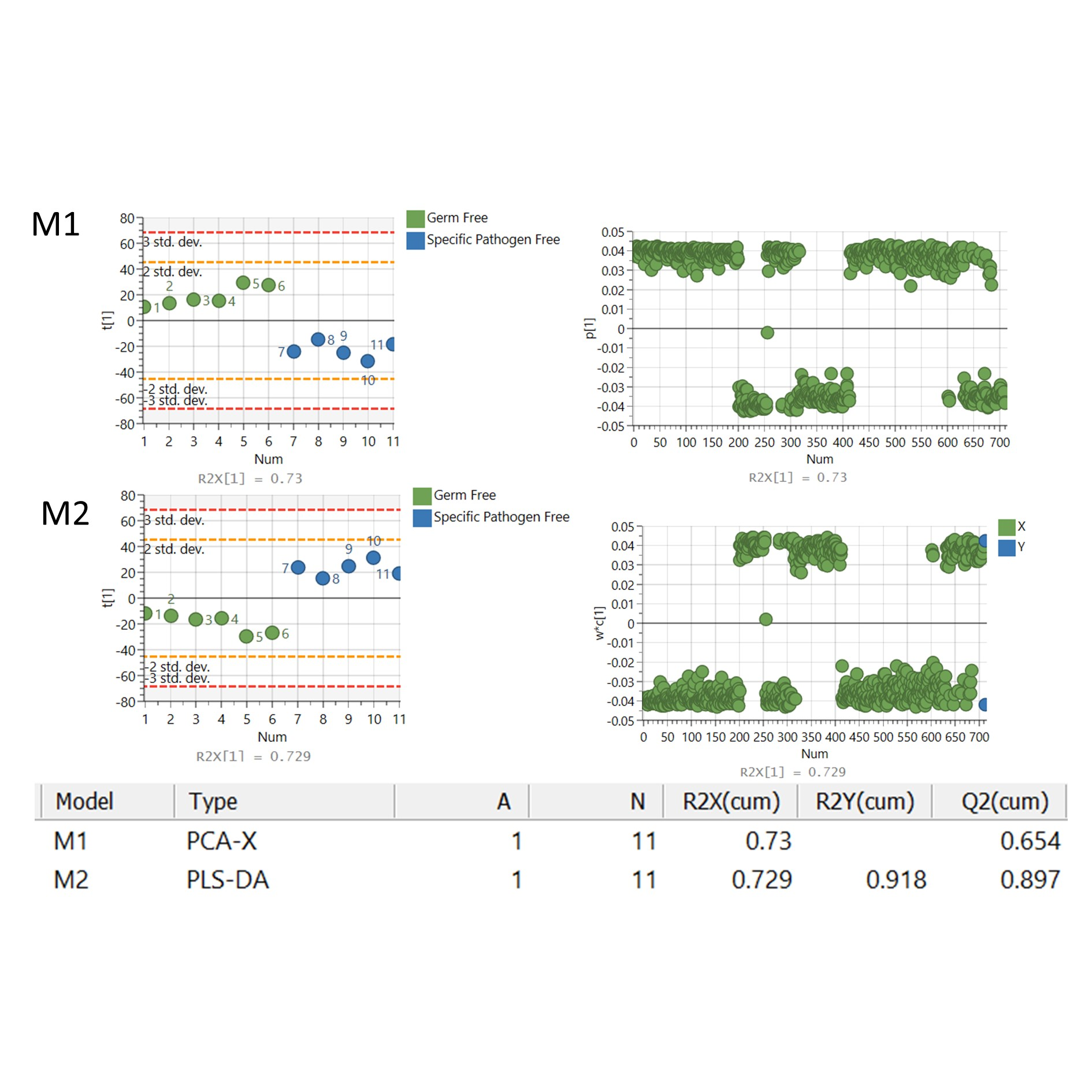


**Figure S2: Unsupervised and supervised discriminant analysis of metabolites in the liver.** M1) Unsupervised PCA analysis was able to discriminate between the groups using metabolite features found in the liver. M2) Supervised PLS-DA analysis also shows that the molecules in the liver can discriminate between groups (GF, green circles; SPF, blue circles). PCA score plots did show distinct clear separation in the liver (parameters: R2X=0.73, Q2=0.654) (Fig. S2). PLS-DA score plot (M2) was also able to separate the groups using the first principle component (parameters: R2X=0.729, R2Y=0.918, Q2=0.897). Specific molecules are not labelled in the corresponding loadings plots due to the large number identified; however, molecules with a VIP>1 are listed in Supplementary Table 3. Analysis was performed using SIMCA 17 software. PLS-DA and PCA score plots consist of component 1 (t [1]).


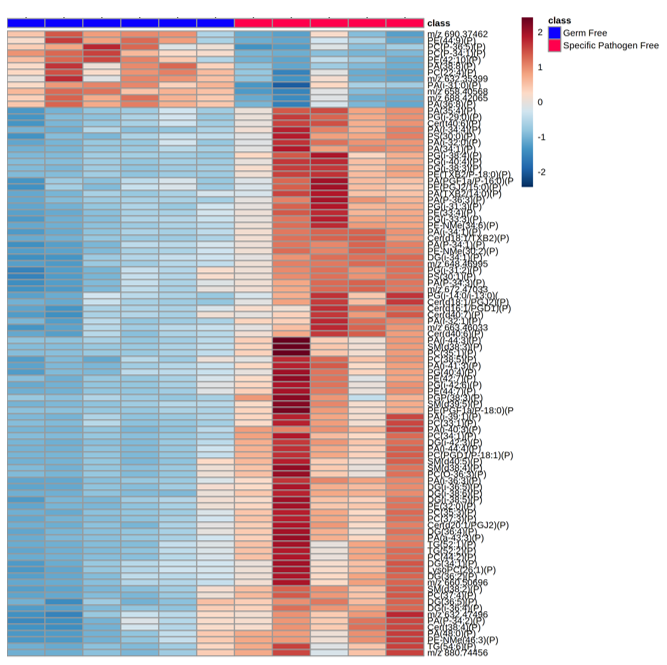


**Figure S3: Heatmap of increased and decreased lipids in liver of GF and SPF mice using positive mode MSI.** Heatmap shows *m/z* of molecules unable to be identified and putatively identified as lipid molecules. Heatmap was created using Metaboanalyst 5.0. The colour of each sample is proportional to the significance of change in metabolite abundance indicated by the colour bar (red, increased; blue, decreased). Rows correspond to metabolites and columns correspond to samples from individual mice (GF, blue, SPF, red).


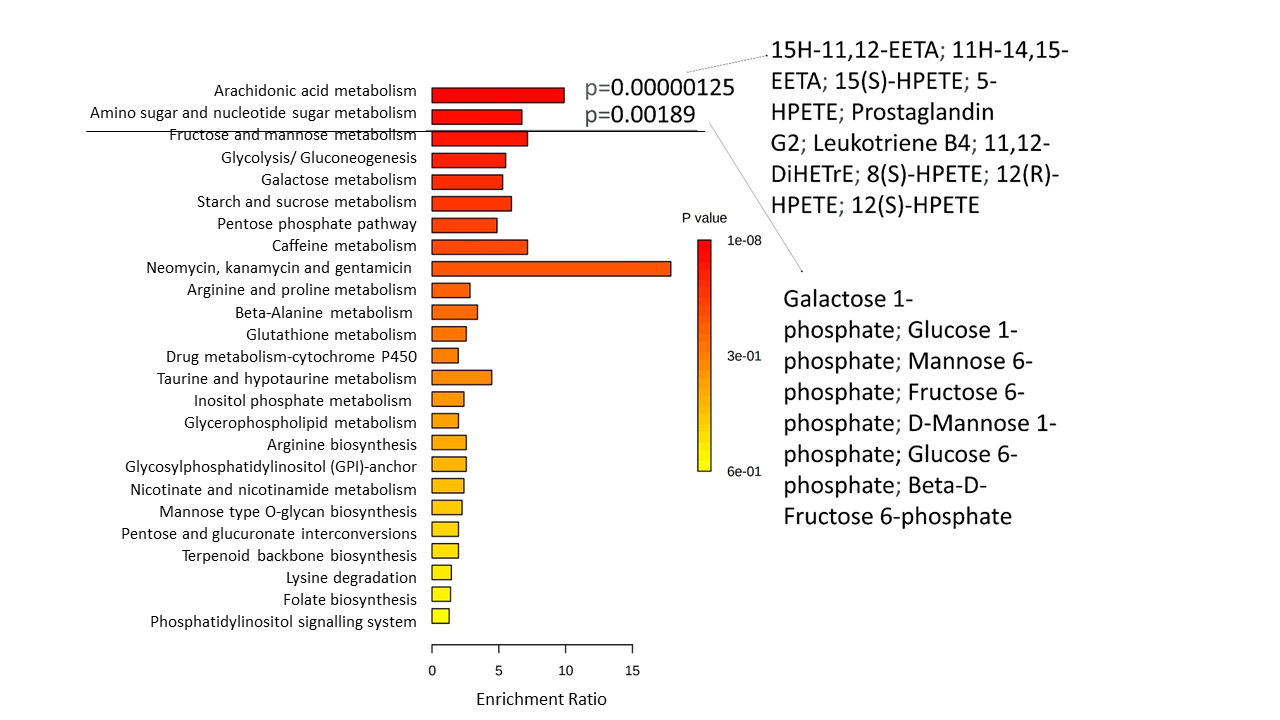


**Figure S4: Enrichment of metabolic pathways in the liver.** Enrichment pathway analysis using KEGG as a reference found molecules involved in at 25 different pathways. The top 2 pathways were significantly enriched, and the molecules involved in the pathway that are present in the dataset are listed.


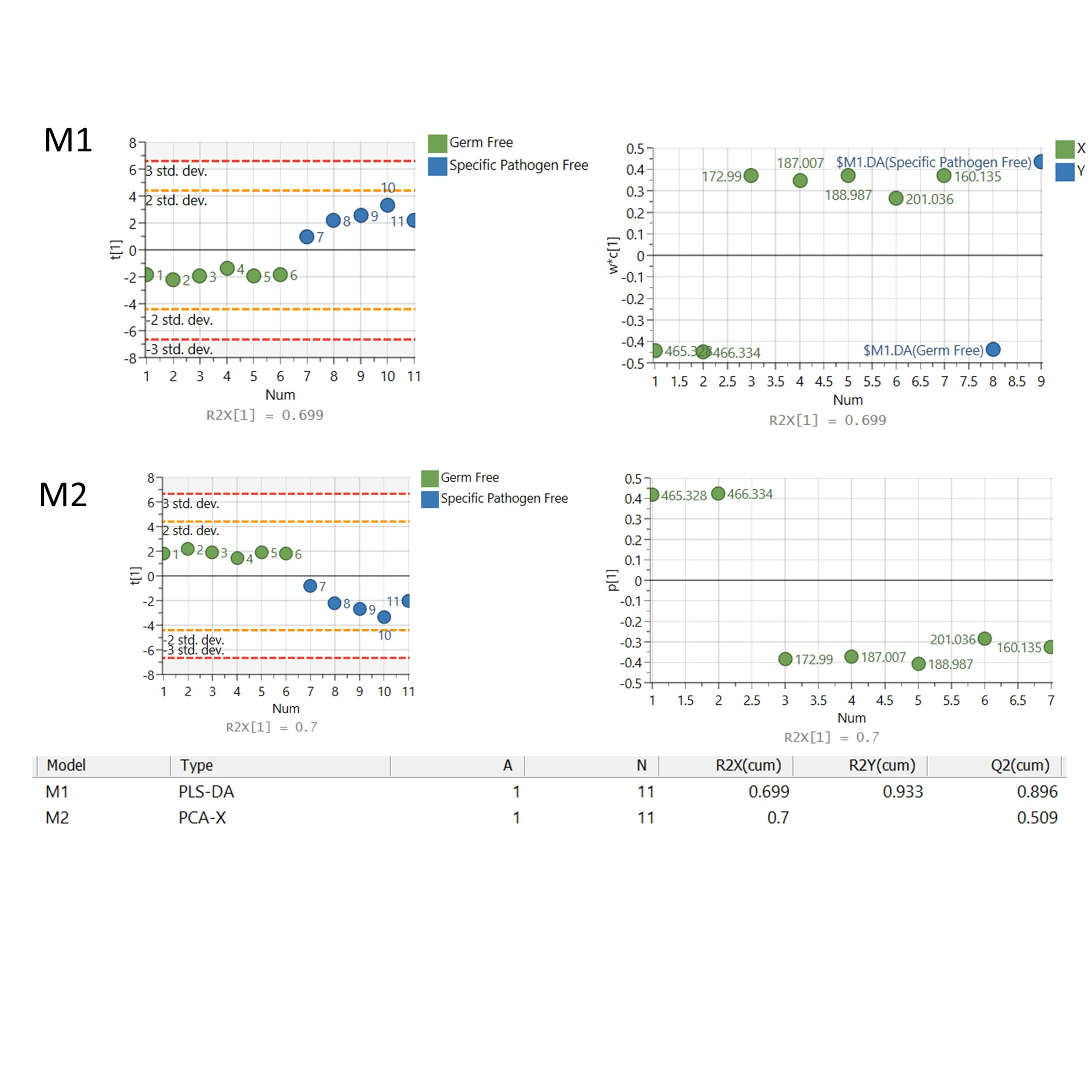


**Figure S5:** **Unsupervised and supervised discriminant analysis of molecules detected in the lung of GF and SPF mice.** M1) Supervised PLS-DA analysis show the molecules in the lung of GF and SPF mice can discriminant between the groups (GF, green circles; SPF, blue circles). M2) Unsupervised PCA analysis was also able to discriminate between the groups using metabolite features. PCA score plots did show clear separation in the lung (parameters: R2X=0.7, Q2=0.509) (Fig. S3). The PLS-DA score plot was also able to separate the groups, as groups are above or below t[1]=0 line (parameters: R2X=0.699, R2Y=0.933, Q2=0.896). Analysis was performed using SIMCA 17 software. PLS-DA and PCA score plots consist of component 1 (t [1]).


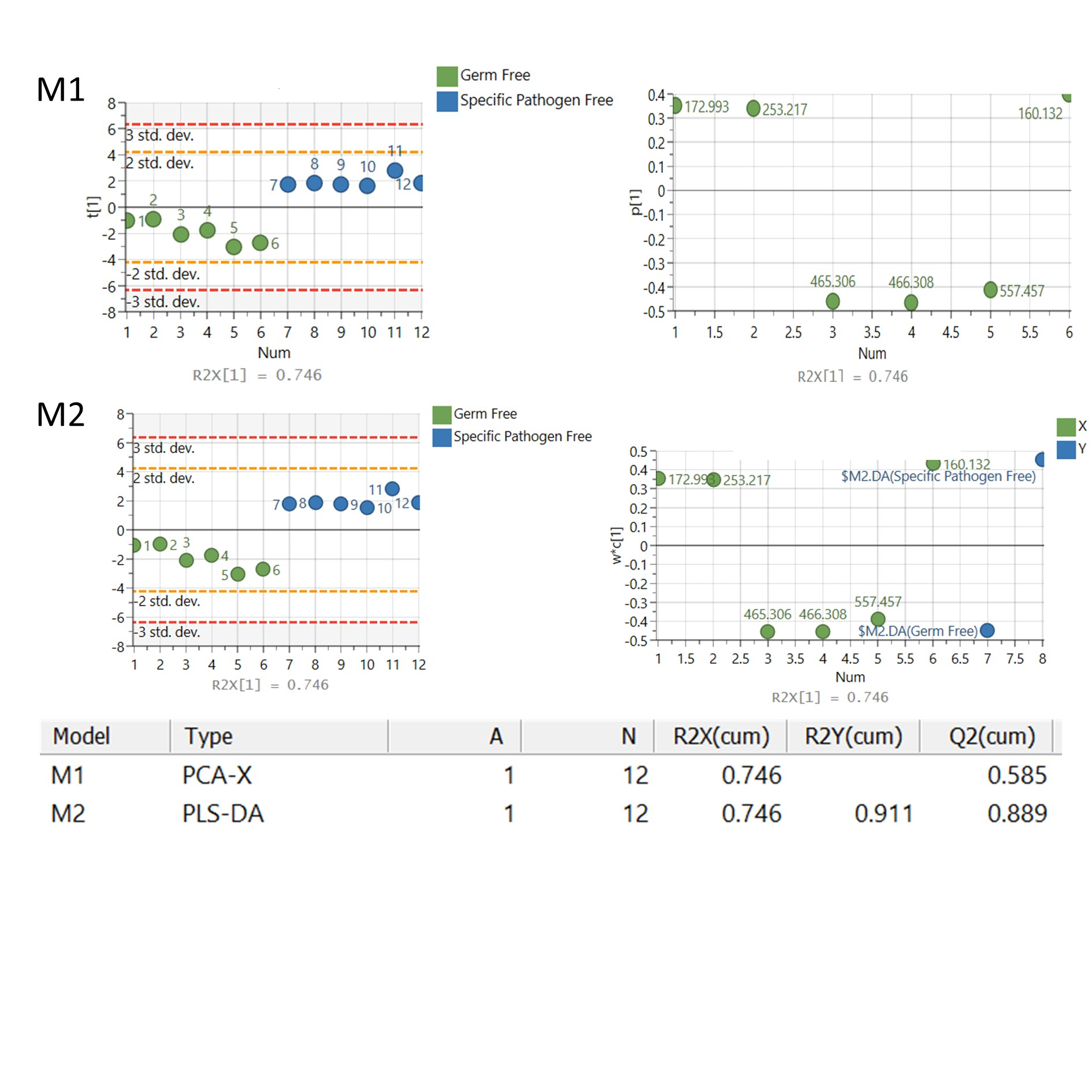


**Figure S6: Unsupervised and supervised discriminant analysis of metabolites found in the spleen.** M1) Unsupervised PCA analysis was able to discriminate between the groups using metabolite features found in the spleen. M2) Supervised PLS-DA analysis also shows that the molecules in the spleen can discriminate between groups (GF, green circles; SPF, blue circles). PCA score plots did show clear separation in the spleen (parameters: R2X=0.76, Q2=0.586). PLS-DA score plot (M2) was also able to separate the groups (parameters: R2X=0.746, R2Y=0.911, Q2=0.889). Analysis was performed using SIMCA 17 software. PLS-DA and PCA score plots consist of component 1 (t [1]).


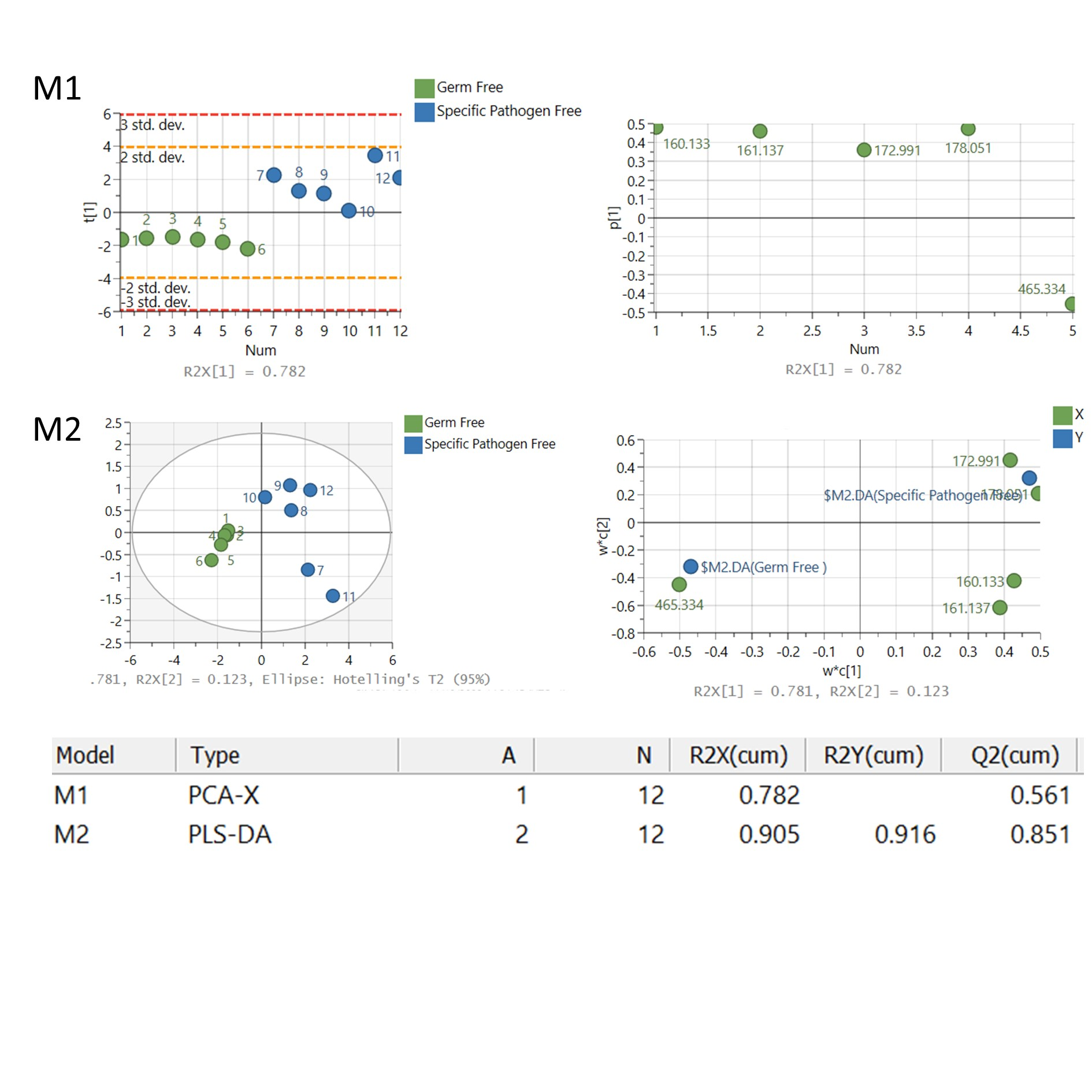


**Figure S7: Unsupervised and supervised discriminant analysis of metabolites found in the kidney.** M1) Unsupervised PCA analysis was able to discriminate between the groups using metabolite features found in the kidney. M2) Supervised PLS-DA analysis shows that the molecules in the kidney can discriminate between groups and contribute to the separation (GF, green circles; SPF, blue circles). PLS-DA score plot (M2) was also able to separate groups based on two significant components, resulting in the groups clustering within an ellipse (parameters: R2X=0.905, R2Y=0.916, Q2=0.851). As both plots had parameters above 0.5, the models are reliably predictive; thus, the metabolic profile in the kidney is able to distinguish SPF from GF group.

Analysis was performed using SIMCA 17 software. PLS-DA score plot consist of components 1 (t [1]) (x-axis) and 2 (t [2]) (y-axis). The ellipse represents the 95% confidence region for Hotelling’s T2 statistic for the model.

**Supplementary Tables**

| **Marker** | **Tag** | **Cell type** | **Clone** | **Tag2** | **Species** | **Dilution** |
| --- | --- | --- | --- | --- | --- | --- |
| ATPase | 141 | Membrane | EP1845Y | 141Pr | Rabbit | V; 1:100 |
| Cleaved caspase 3 | 142 | Apoptosis | D3E9 | 142Nd | Rabbit | V; 1:25 |
| Vimentin | 143 | Mesenchymal cells | D21H3 | 143Nd | Rabbit | V; 1:400 |
| B220 | 144 | B cells | RA3-6B2 | 144Nd | Rat | V; 1:25 |
| CD68 | 145 | Macrophages | FA-11 | 145Nd | Rat | V; 1:100 |
| CD31 | 146 |  |  | 146Nd |  | V; 1:50 |
| CD45 | 147 | Myeloid cells | 30-F11 | 147Sm | Rat | V; 1:100 |
| PanCK | 148 | Epithelial cells | C11 | 148Nd | Mouse | V; 1:200 |
| CD19 | 149 | B cells | 6D5 | 149Sm | Rat | V; 1:50 |
| CD103 | 150 | Dendritic cells | AF1990 | 150Nd | Goat | V; 1:25 |
| Ly6G | 151 |  | 1A8 | 151Eu | Rat | V; 1:50 |
| PAKT | 152 |  | D9E | 152Sm |  | V; 1:25 |
| CD11c | 153 | Dendritic cells | D1V9Y | 153Sm | Rabbit | V; 1:50 |
| CD11b | 154 | Macrophage subset | M1/70 | 154Sm | Rat | V; 1:50 |
| F4/80 | 155 | Macrophages | CI:A3-1 | 155Gd | Rat | V; 1:50 |
| CD163 | 156 | M2 Macrophages | TNKUPJ | 156Gd | Rat | V; 1:50 |
| e cadherin | 158 | Epithelial cells | 2.40E+11 | 158Gd | Rabbit | V; 1:100 |
| Collagen 1 | 159 |  | Poly | 159 Tb |  | V; 1:200 |
| glut1 | 160 | Hypoxia | EPR3915 | 160Gd | Rabbit | V; 1:100 |
| CD69 | 161 |  | Poly | 161Dy |  | V; 1:400 |
| Ki67 | 162 | Proliferation | B56 | 168Er | Mouse | V; 1:100 |
| a-SMA | 163 | Fibroblast and pericyte phenotype | Polyclonal | 163Dy | Rabbit | V; 1:400 |
| lyve1 | 164 | lymphatic vessels | poly | 164Dy | Rabbit | V; 1:300 |
| FOXP3 | 165 | Treg | FJK-16s | 165Ho | Rat | V; 1:25 |
| epcam | 166 | Epithelial cells | G8.8 | 166Er | Rat | V; 1:900 |
| NKp46 | 167 | NK cells | Polyclonal | 147Sm | Goat | V; 1:25 |
| CD8 | 168 | T cells (CD8+) | 53-6.7 | 146Nd | Rat | V; 1:50 |
| CD206 | 169 | M2 Macrophages | CD68C2 | 169Tm | Rat | V; 1:50 |
| arg1 | 170 | M2 Macrophages | Poly | 170Er | Sheep | V; 1:100 |
| CD4 | 172 | T cells (CD4+) | RM4-5 | 172Yb | Rat | V; 1:50 |
| MHCII | 174 | Immune cells | M5/114.15.2 | 174Yb | Rat | V; 1:200 |
| Granzyme B | 176 | Activated T cells | Polyclonal | 176Yb | Goat | V; 1:300 |
| Collagen IV | 209 | ECM | Polyclonal | 209Bi | Rabbit | V; 1:100 |

**Supplementary Table 1:** List of immune cell marker antibodies, clone and tags used for all IMC staining.

| **Phenotype** | **Markers** |
| --- | --- |
| Helper T cells | CD3 + CD4 |
| Cytotoxic T cells | CD3 + CD8 |
| Regulatory T cells (Tregs) | CD3 + CD4 + FOXP3 |
| Activated Cytotoxic T cells | CD3 + CD8 + Granzyme B |
| Natural killer cell | NKp46 |
| Activated natural killer cell | NKp46 + Granzyme B |
| B cells | B220 + CD19 |
| Neutrophils | Ly6G + CD11b |
| Dendritic cells | CD11c + M1 MHCII |
| Antigen-presenting Dendritic cells | CD11c + MHCII + CD103 |
| Macrophages | F4/80 + CD68 + CD11b (some overlap with dendritic cells) |
| M2c Macrophages | F4/80 + CD163 |
| M2a/c Macrophages | F4/80 + Arg1 |
| M2a Macrophages | F4/80 + CD206 |
| M1 and M2b Macrophages | F4/80 |
| M1 Macrophages | F4/80 + MHCII |
| Blood vessel | CD31 |
| Pericyte (found around large blood vessels) | aSMA |
| Mesenchymal cell | Vimentin |
| Epithelial cell | E Cadherin + PanCK + EpCam |
| Proliferation | Ki67 |
| PI3K signalling | pAKT |
| Hypoxia | GLUT1 |
| Apoptosis | CC3 |

**Supplementary Table 2:** Immune cell phenotypes defined by specific markers in IMC analysis.

Figure 1

Figure 3

Figure 6

**Supplementary Table 3:** Details of statistical tests for graphs in Figures 1, 3 and 6.
